# Modelled responses of the Kalahari Desert to 21^st^ century climate and land use change

**DOI:** 10.1038/s41598-017-04341-0

**Published:** 2017-06-20

**Authors:** Jerome R. Mayaud, Richard M. Bailey, Giles F. S. Wiggs

**Affiliations:** School of Geography and the Environment, Oxford University Centre for the Environment, University of Oxford, Oxford, OX1 3QY UK

## Abstract

Drylands are home to over 2 billion people globally, many of whom use the land for agricultural and pastoral activities. These vulnerable livelihoods could be disrupted if desert dunefields become more active in response to climate and land use change. Despite increasing knowledge about the role that wind, moisture availability and vegetation cover play in shaping dryland landscapes, relatively little is known about how drylands might respond to climatic and population pressures over the 21^st^ century. Here we use a newly developed numerical model, which fully couples vegetation and sediment-transport dynamics, to simulate potential landscape evolution at three locations in the Kalahari Desert, under two future emissions scenarios: stabilising (RCP 4.5) and high (RCP 8.5). Our simulations suggest that whilst our study sites will experience some climatically-induced landscape change, the impacts of climate change alone on vegetation cover and sediment mobility may be relatively small. However, human activity could strongly exacerbate certain landscape trajectories. Fire frequency has a primary impact on vegetation cover, and, together with grazing pressure, plays a significant role in modulating shrub encroachment and ensuing land degradation processes. Appropriate land management strategies must be implemented across the Kalahari Desert to avoid severe environmental and socio-economic consequences over the coming decades.

## Introduction

## Drylands as non-equilibrium environments

Dryland environments are extreme in their nature, typified by non-equilibrium conditions in climate, vegetation and geomorphology^1–3^. The strong interannual variability in precipitation that is characteristic of drylands^4^ often results in the formation of dynamic vegetation patterning^5^. These patterns, which range from ‘gaps’ to ‘labyrinths’, ‘stripes’ and ‘spots’, likely arise from adaptation to resource limitation (specifically of water), with plant-plant facilitation occurring at short distances and competition acting at larger distances^6–9^. In turn, the patchy nature of semi-arid vegetation makes it an important variable in shaping the rate and extent to which geomorphological processes operate, notably wind-blown sediment movement^10, 11^.

The interactions between vegetation, geomorphology and, increasingly, humans, often result in shifts in the composition and patterning of dryland ecosystems. Ecosystem regime shifts involve the transition from one stable state to an alternative stable state^12–15^, and can broadly be divided into two categories: (i) small, slow and reversible; and (ii) large, abrupt and irreversible. Large, abrupt shifts can be detrimental to dryland ecosystems because they cause rapid and widespread loss of bioproductivity and biodiversity^16, 17^. This affects ecosystem function and stability^18, 19^ and thus a broad range of ecosystem services, from economic resources to recreation, firewood and food^17, 20^, which help to sustain a large majority of dryland livelihoods. There is particular concern about how land degradation, particularly through bush encroachment and associated losses of grass species^8, 21–24^, may intersect with increasing rural poverty levels^25^. Whilst large-scale vegetation shifts are known to result from variations in external (climatic and CO_2_) forcing^26^, the potential impacts of 21^st^ century climatic change on dryland vegetation cover and composition remain unclear^27^.

Abrupt ecosystem shifts in drylands can also be triggered or exacerbated by a range of human disturbances, such as agriculture, grazing and fire^28–32^. Modelling studies have shown that such disturbances can lead to hysteretic ecosystem recovery^6, 16, 33^, whereby the state of the landscape depends on past as well as current conditions. The potential existence of vastly different vegetation covers in the same climatic context has important implications for sediment movement and subsequent desertification processes^34, 35^. Ultimately, the combination of pressures from climate change and human activities could lead to large-scale reactivation of dune fields and desert encroachment in many of the world’s most vulnerable regions^36, 37^. However, the interactions of fire, grazing and factors affecting ecogeomorphic tipping points are not well understood^1, 3, 38^. It is therefore vitally important to model dryland systems in a holistic way, by emphasising the complex coupling between vegetation and sediment transport dynamics.

Here, we explore the potential impacts of climate change and human land management (fire and grazing) on landscape processes in the Kalahari Desert in southern Africa. For this we use a coupled cellular automaton, the Vegetation and Sediment TrAnsport model (ViSTA)^39^. ViSTA explicitly simulates known feedbacks between vegetation growth, wind flow dynamics and sediment flux over a surface (see Methods). Analysable outputs from the model include vegetation cover, proportions of different vegetation types (grasses, shrubs and trees) and sediment transport levels, which help to indicate land degradation processes. Mayaud *et al*.^39^ provide an extensive set of verification tests for ViSTA, with empirical data supporting the model formulation as far as possible.

**Figure 1:**
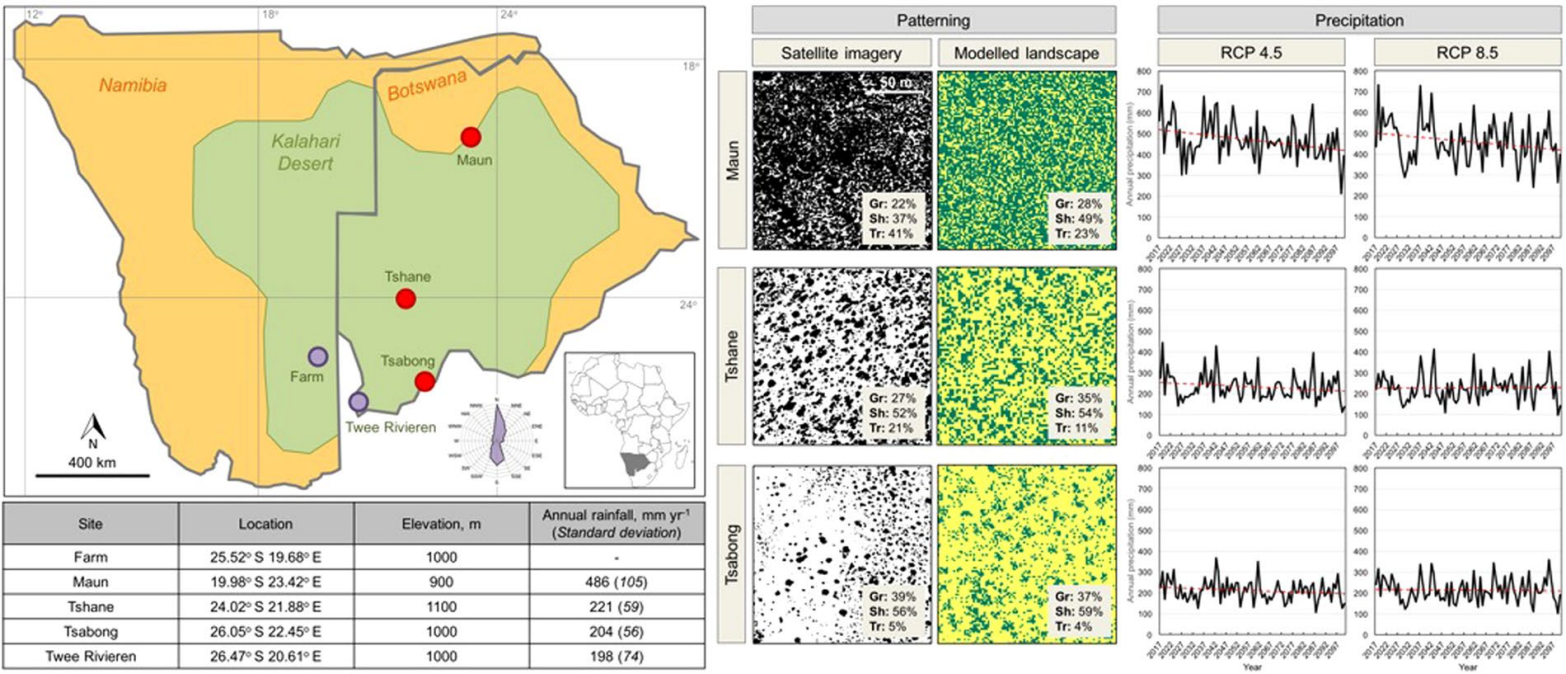
Map: Locations of experimental sites (main analysis sites shown as red dots, additional sites shown as purple dots). Green shaded area shows the extent of the Kalahari Desert in Botswana and Namibia. Wind rose shows aggregated mean monthly wind directions over the period Jan 1994–Dec 2013 at Twee Rivieren. Map was drawn digitally in MS Powerpoint (v.2010) by J.R. Mayaud, based on satellite data from Google Earth (v.5.1). Modelled vegetation states for the three study sites (for 2000, RCP 4.5) are compared to satellite imagery (Google Earth, v.5.1; converted to black (vegetation present) and white (bare surface) using a visually determined threshold). Proportions of vegetation types, both modelled and measured along vegetation transects near each site (see Supplementary Information for methods), are shown in inset grey boxes. Maun (19.99°S, 23.69°E) is located on the edge of the Okavango Delta, with a dominant grass/shrub system and some dense tree cover. Tshane (24.13°S, 22.04°E), in the middle of the Kalahari Desert, is characterised by lower cover of mixed grasses and shrubs, with occasional trees. Tsabong (26.04°S, 22.21°E) in southern Botswana also has low vegetation cover, mainly composed of grasses and shrubs with isolated trees. Graphs show projected changes in annual precipitation over the period 2017–2100, for Maun, Tshane and Tsabong, under the RCP 4.5 and RCP 8.5 scenarios. Dotted red lines show linear best fit trends. Table shows mean observed annual rainfall over the period Jan 1984–Dec 2013 (standard deviations in brackets show interannual variability).

For our modelling experiments, we chose three sites along a North-South rainfall gradient across the Kalahari (Maun, Tshane and Tsabong; see Fig. 1), to provide a variety of current environments and projected climatic changes. Although the southern African dune system is largely inactive today, it developed over the course of multiple episodes of aridity since the last interglacial period^40, 41^, and is likely to experience enhanced dune activity in the coming decades^36^. We used two of the IPCC’s^42^ main emissions scenarios (‘representative concentration pathways’, RCPs) to derive projected climate changes at our study sites: RCP 4.5, representing stabilisation of greenhouse gas (GHG) emissions (to 580 ppm CO_2_-equivalent by 2100), and RCP 8.5, a high GHG emission scenario (to 1230 ppm CO_2_-equivalent by 2100).

## Kalahari futures

The current climate of southern Africa is characterised by marked interannual and interdecadal variability^43^, due to influence from both tropical and mid-latitude climate systems^44^. Against this significant interannual variability, precipitation is projected to decrease at all three sites over the 21^st^ century, in both the RCP 4.5 and RCP 8.5 scenarios (see graphs in Fig. 1). There are clear upward trends in temperature at all sites, although their magnitude differs between RCP scenarios (see Supplementary Note 2 and Supplementary Figure 2). In this study, we focus on precipitation as the key driving factor for vegetation change in the ViSTA model. We consider the uncertainties in the processes described by the model, as well as the uncertainties in climate projections, to robustly explore potential futures for the Kalahari Desert.

**Figure 2:**
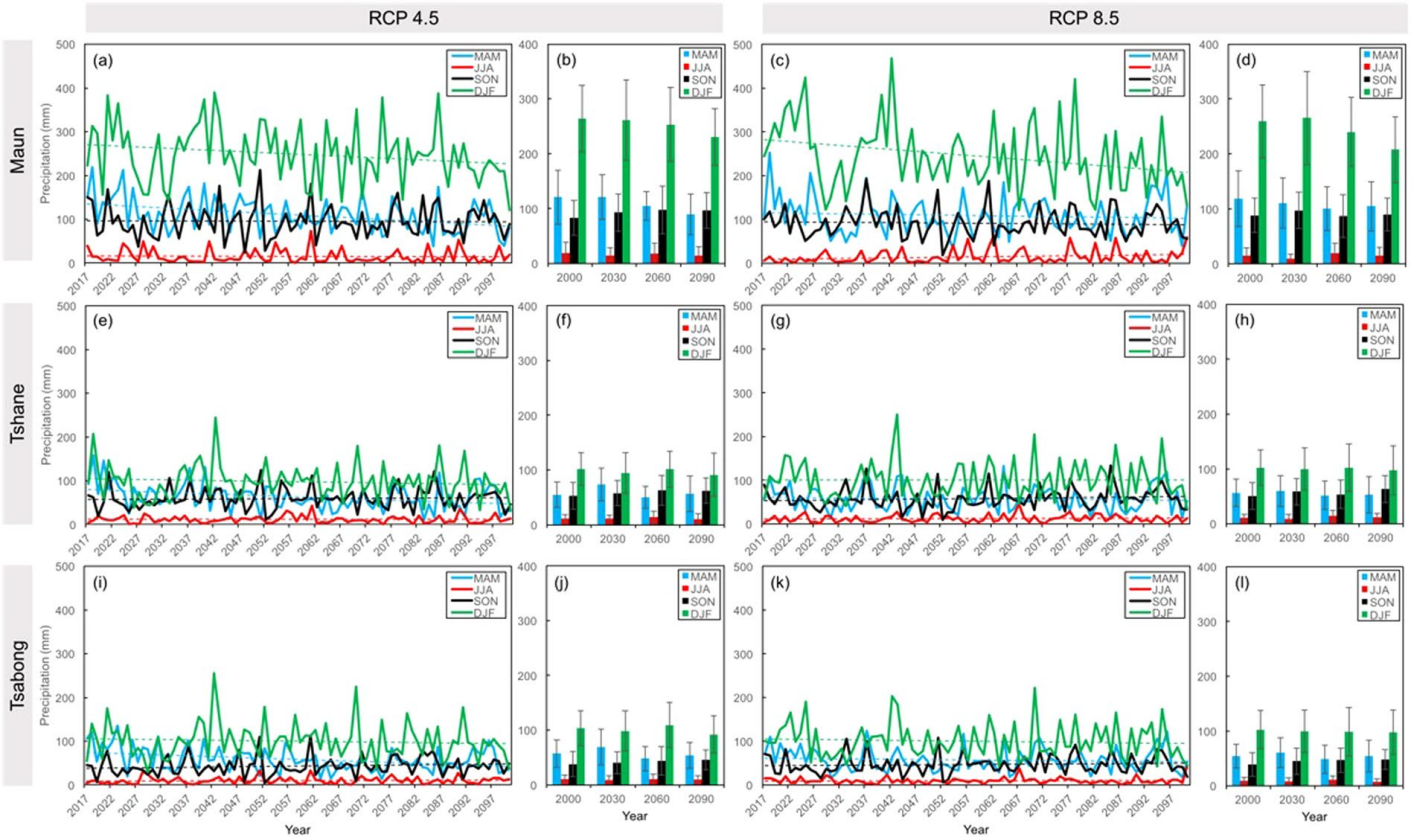
(**a**,**c**,**e**,**g**,**i**,**k**) Projected changes in precipitation, separated by season, over the period 2017–2100 at each study site. Dotted lines show linear best fit trends; (**b**,**d**,**f**,**h**,**j**,**l**) mean total precipitation in each season, for the chosen years (2030, 2060, 2090) and baseline year (2000). Error bars show standard deviations.

Projected seasonal changes in precipitation are shown in Fig. 2. At Maun, there is a clear signal of increasingly dry winters (JJA), which are thought to result from poleward-shifting storm tracks^45, 46^. However, drier winters may have limited societal impact because this is already a very dry season^46, 47^. At Tshane and Tsabong, increased winter (JJA) aridity in the first half of the century does not appear to contribute to subsequently drier spring periods (SON), as has been previously hypothesized^45, 48^.

**Figure 3:**
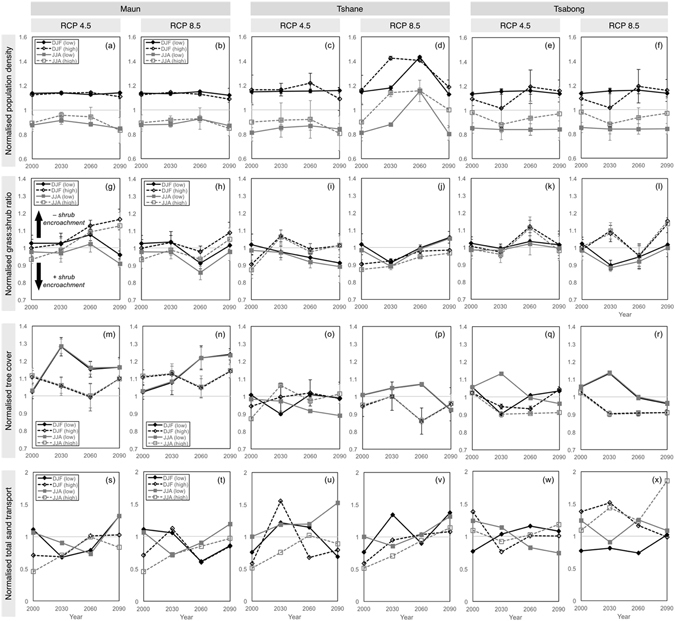
Equilibrium landscape characteristics at the three study sites for the three chosen years (2030, 2060 and 2090), separated by wet summer season (DJF) and dry winter season (JJA), and normalised to the annual average for the baseline year (2000, RCP 4.5). Results are shown for RCP 4.5 and RCP 8.5 scenarios, in low variability (standard deviation, $\sigma$ = 30 mm annual equivalent) and high variability ($\sigma$ = 160 mm annual equivalent) precipitation conditions. Landscape characteristics: (**a**–**f**) normalised population density; (**g**–**l**) normalised grass:shrub ratio, where values above unity represent decreased shrub encroachment; (**m**–**r**) normalised tree cover; (**s**–**x**) normalised total sediment transport across the domain. Model was run to equilibrium for the equivalent of 200 model years, with 20 wind events every 3 months, over a grid of 150 × 150 cells. The domain was randomly initiated with 90% vegetation cover and random sediment depth. Landscape characteristics were calculated as means over the last 10 years of each run. Error bars show coefficients of variation where appropriate.

For each study site, the ViSTA model was run to equilibrium with the projected conditions in three key years (2030, 2060 and 2090) and a ‘baseline’ year (2000) (Fig. 2b,d,f,h,j,l), using a low-variability and high-variability scenario (see Methods). Figure 3 displays certain landscape statistics at equilibrium separated by wet season (DJF) and dry season (JJA), normalised to annual statistics for the baseline year. We use the term ‘population density’ to refer to the overall number of plant-covered cells relative to the total grid size. The modelled equilibrium vegetation patterning states compare well with satellite imagery and vegetation transects at each study site (see Fig. 1). The model replicates the high vegetation cover at Maun, with numerous trees amongst grass/shrub savanna. The model also simulates generally patchier cover at Tshane and Tsabong, with lower tree proportions and higher shrub and grass proportions than at Maun.

At Maun and Tsabong, population densities stay roughly stable throughout the 21^st^ century, with few changes between RCPs, key years or variability scenarios (Fig. 3a,b,e,f). At Tshane, population density increases by up to 30% around 2030 and 2060 (Fig. 3c,d), particularly under RCP 8.5, due to an increase in spring (SON) and summer (DJF) rainfall (Fig. 2h). In terms of grass:shrub ratios, high variability scenarios generally tend to result in reduced shrub encroachment (Fig. 3g–l), because more variable rainfall favours the establishment of pioneer grass species. RCP scenarios also influence grass:shrub ratios. For instance, in the latter half of the century Tshane experiences greater shrub encroachment under RCP 4.5 compared with RCP 8.5. This occurs because drier summers (DJF) under RCP 8.5 allow grasses to compete more favourably with shrubs, as shown by changes in average plant ages (Supplementary Figure 9), and by the fact that grass:shrub ratios increase as DJF rainfall is ramped up (Supplementary Note 6 and Supplementary Figure 11a). At Maun, proportional tree cover generally increases during both wet and dry seasons as the century progresses, particularly in low-variability scenarios (Fig. 3m,n). At Tshane and Tsabong, proportional tree cover may decrease slightly over the century, remaining higher in low-variability scenarios than in high-variability scenarios, particularly under RCP 8.5 (Fig. 3p,r).

The vegetation dynamics result in different sediment movement conditions at each site. In the ViSTA model, the strength of the feedbacks between sediment movement and vegetation growth can be modified to suit the parameterisation needed for different plant types and environments (Supplementary Note 6 and Supplementary Figure 10). Maun experiences a general increase in sediment movement in both seasons throughout the century (Fig. 3s,t), although sediment movement tends to decrease in the wet season (DJF) under RCP 8.5. Tshane also experiences increased sediment movement throughout the century (Fig. 3u,v), particularly under RCP 8.5, despite an increase in general vegetation cover. This is attributed to greater shrub encroachment during this period, since porous shrubs tend to provide less extensive sheltering effects than the bluff bodies of grasses^49^. An additional experiment (Supplementary Note 6 and Supplementary Figure 11b) demonstrates that a landscape populated with low-porosity elements experiences lower sediment transport rates than one populated with high-porosity elements. Sediment movement patterns are less clear at Tsabong (Fig. 3w,x), although there is the potential for much greater transport during winter in a high-variability scenario under RCP 8.5.

**Figure 4:**
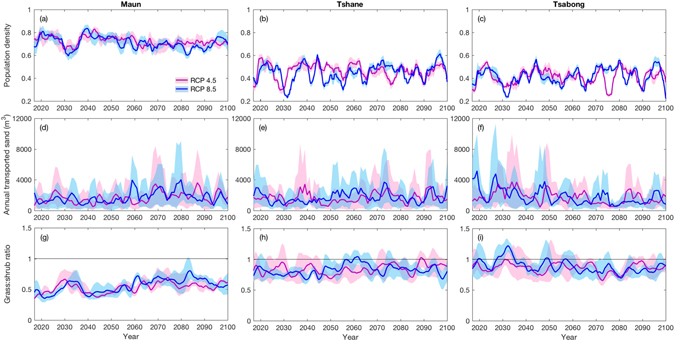
Landscape characteristics during the transient model runs, from 2017 to 2100, at the three study sites. Results are shown for RCP 4.5 (pink) and RCP 8.5 (blue) scenarios. Landscape characteristics: (**a**,**b**,**c**) population density; (**d**,**e**,**f**) total annual sand transport across the domain; (**g,h,i**) grass:shrub ratio, where values above unity represent grass dominance, and values below unity represent shrub dominance. Model was run 10 times per site and RCP from the year 1960, using projected precipitation data with random variation based on 5-year moving averages of seasonal standard deviations. 20 wind events occurred every 3 months, over a grid of 150 × 150 cells. The domain was randomly initiated with vegetation cover and species proportions observed at the end of an equilibrium run for 1960. Landscape characteristics are shown as three-year moving averages (solid coloured lines). Shaded area shows the range of model output across the 10 model runs for each site and RCP.

Transient experimental runs were also performed (see Methods), because dryland landscape responses are path-dependent and can be hysteretic in nature^6, 39^. Figure 4 displays landscape characteristics as they change continuously through the century. At Maun, vegetation population density generally declines as the century progresses under both RCPs (Fig. 4a), with simultaneous trends of increasing sediment movement (Fig. 4d) and decreasing shrub dominance (Fig. 4g). In the early 2030s, a drop in population density, accompanied by a spike in the grass:shrub ratio, can be linked to a significant drop in summer (DJF) precipitation (Fig. 2a). At Tshane and Tsabong, population density remains lower and more variable than at Maun (Fig. 4b,c), with no discernible directional trend. Population density at Tshane and Tsabong shows greater variability because of the system’s proximity to collapse at these locations, owing to generally low precipitation levels. This leads to higher grass proportions than at Maun (Fig. 4h,i), with Tsabong experiencing outright grass dominance in the early 2030s as a result of the summer precipitation decline. This may reduce sediment movement and be more amenable to livestock grazing, but could also promote greater fire propagation^32, 50^. Sediment movement at Tshane remains broadly stable throughout the century (Fig. 4e), whilst at Tsabong sediment movement tends to be highest in the first half of the century (Fig. 4f).

## The impact of fire and grazing

**Figure 5:**
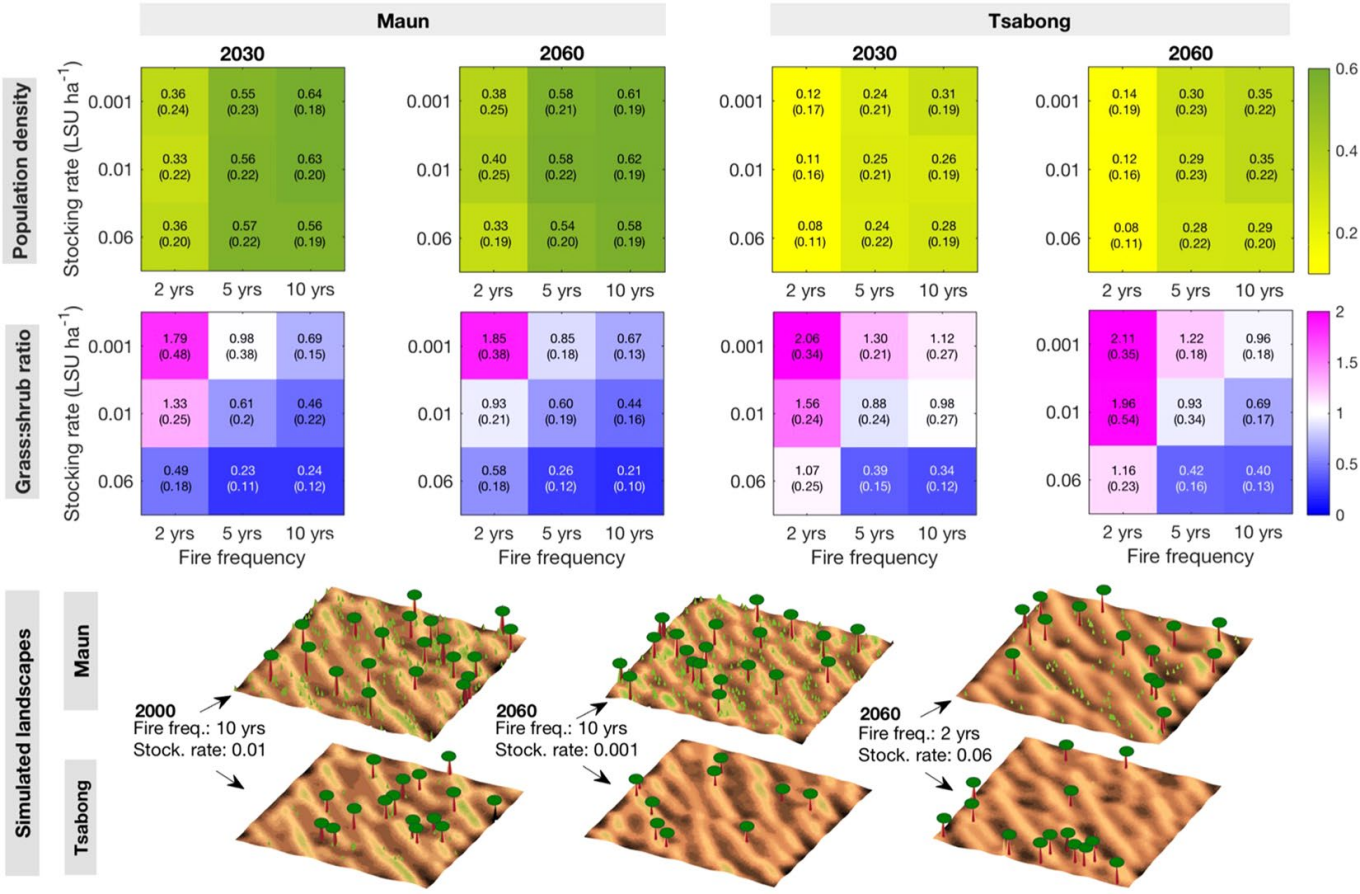
Matrices of population density and grass:shrub ratios at Maun and Tsabong under RCP 4.5, for 2030 and 2060, with three different fire frequencies (low: once every 10 years, medium: once every 5 years, high: once every 2 years) combined with three different grazing pressures (low stocking rate: 0.001 LSU ha^−1^, medium: 0.01 LSU ha^−1^, high: 0.06 LSU ha^−1^) imposed on the landscape (see Methods). Values are given in each matrix square (standard deviation in brackets), with colour scaling. Three-dimensional examples of simulated landscapes in 2000 and 2060 are displayed for comparison purposes (trees shown as trunks with distinct crowns, shrubs/grasses shown as green patches). Values above grass:shrub unity represent grass dominance, and values below unity represent shrub dominance. Model was run 3 times per fire/grazing scenario at both sites from the year 1960, using projected precipitation data with random variation based on 5-year moving averages of seasonal standard deviations. 20 wind events occurred every 3 months, over a grid of 150 × 150 cells. The domain was randomly initiated with vegetation cover and species proportions observed at the end of an equilibrium run for 1960. Landscape characteristics in the matrices were calculated as means over the 10 years around both chosen dates.

To explore the added impact of human management on the Kalahari Desert system, grazing and periodic fires were imposed in the ViSTA model. Low, medium and high grazing was simulated (based on typical animal stocking rates for Kalahari rangelands), in combination with low, medium and high fire frequencies (see Methods). Simulations for Maun and Tsabong were performed, as these represent the climatic extremes of our sites, under RCP 4.5 only. To focus our results on the relevant scale of a human lifetime, we present results for 2030 and 2060 in Fig. 5.

At both Maun and Tsabong, the biggest impact on vegetation population density is fire frequency, with density decreasing by up to 48% at high fire frequencies compared to low frequencies (Fig. 5). Grazing pressure has a secondary impact, with up to 43% decrease in density between low stocking rate and high stocking rate. In the worst-case scenarios, population density may decrease to 8% at Tsabong. This could lead to important dune reactivation, as evidence shows that sand transport increases dramatically below a threshold of 12–15% vegetation cover^51, 52^.

Both fire frequency and stocking rate have a notable impact on grass:shrub ratios at the two sites (Fig. 5). Ratios decrease (i.e. shrubs increasingly dominate) by up to 68% at low fire frequencies compared to high frequencies, and ratios decrease by up to 77% at high grazing pressures compared to low grazing pressures. Frequent fire occurrence leads to a relative increase in short-lived grass populations because grasses preferentially recolonise bare areas^53, 54^. Longer fire cycles allow shrubs to more readily establish and increasingly out-compete grasses. The establishment of shrub communities is also enabled by the generally higher precipitation levels at Maun. At Tsabong, the harsher precipitation conditions ensure that grasses dominate the system when grazing is low and/or fires are frequent. In the worst-case scenarios, grass:shrub ratios could decline to 0.21 at Maun and 0.34 at Tsabong, representing severe shrub encroachment. Such a shrub-dominated state would persist through amplifying feedbacks linked to the fire regime^55, 56^. This could result in a substantial increase in bare soil fractions and subsequent enhancement of soil erosion rates in barren patches^57–59^.

These findings corroborate oral history evidence of vegetation and dune dynamics in the southwest Kalahari (see Supplementary Note 4). Over the last thirty years, the region has experienced increased agricultural intensification and fence construction, which has led to greater grazing pressures and severe shrub encroachment in some areas^22, 24, 25^. Shrub encroachment can be combatted using ‘debushing’ herbicides (Supplementary Figure 5), but this remains expensive to deliver over large areas^60^. Much heavily grazed farmland in the southwest Kalahari now consists of bare, intermittently active dune crests^61^, which have migrated noticeably within a human lifetime^24, 62^.

It is difficult to define optimal vegetation and soil conditions for agriculture and pastoral livelihoods^60^. For instance, shrub encroachment is often cited as having negative impacts on pastoral activities^27, 63^, yet over the short-term land managers perceive certain shrub species to be important windbreak and forage resources^22, 23^. Furthermore, the extent to which shrub-encroached land can be considered ‘degraded’ depends heavily on shrub traits^64^. At Tshane and Tsabong, decreased shrub encroachment under some scenarios (Fig. 3j–l) could ameliorate conditions for cattle, and the associated decrease in sediment movement (e.g. Fig. 4j) may help to improve soil fertility^65^. However, a decrease in proportional tree cover (Fig. 3p–r) at these locations could negatively affect other ecosystem services such as firewood provision and cultural usages^17, 38^.

**Figure 6:**
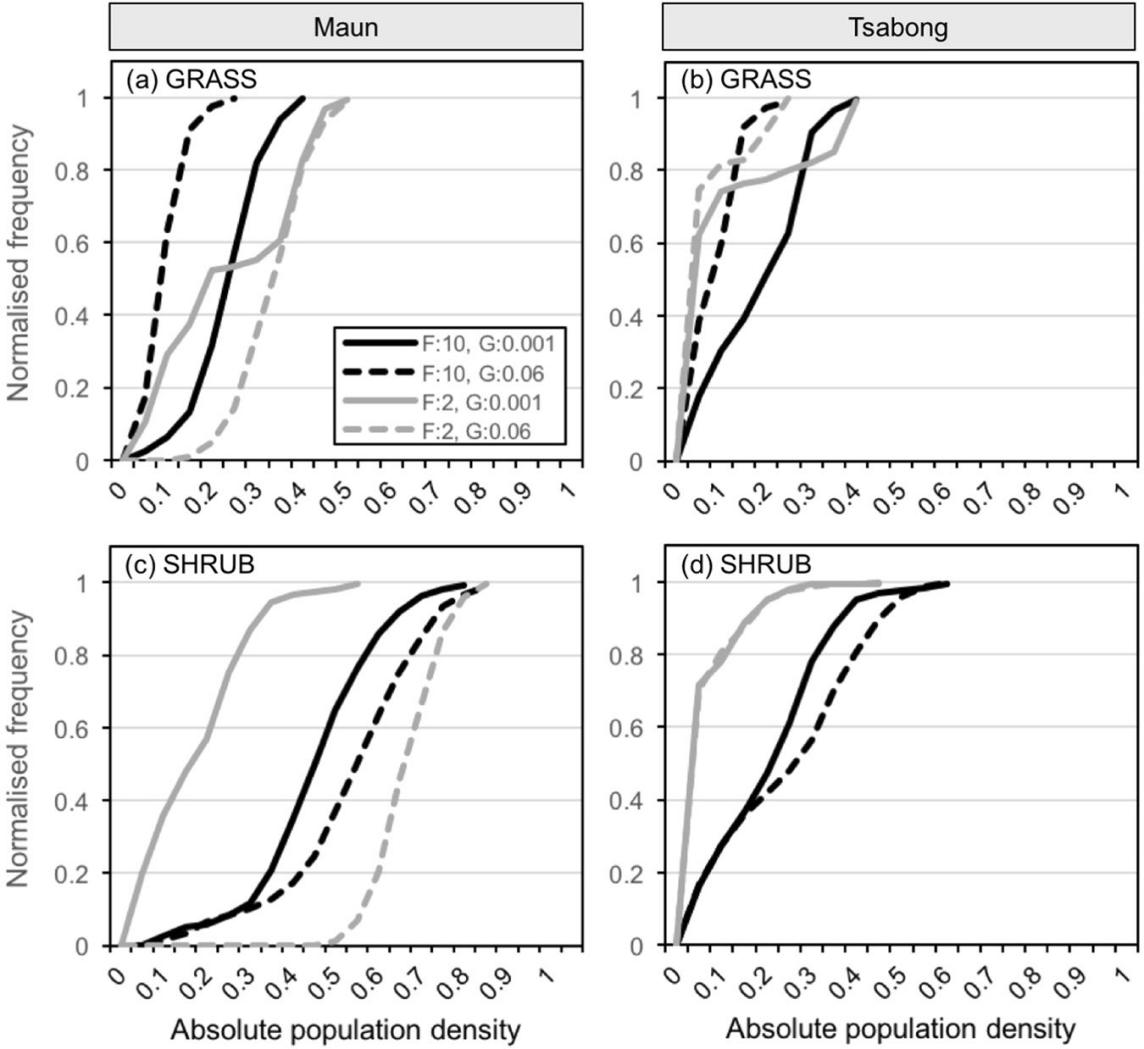
Cumulative frequency curves of absolute population density of grasses and shrubs at Maun and Tsabong, over the period of 2017–2100, for different fire/grazing scenarios. Data are from the same model runs presented in Fig. 5. Curve data represents averaged density-frequency data (every 3 months) from the 3 model runs for each fire/grazing scenario. Four management scenarios are presented: low fire and low grazing (fire: every 10 years, grazing: 0.001 LSU ha^−1^; solid black line), low fire and high grazing (f: 10, g: 0.06; dotted black line), high fire and low grazing (f: 2, g: 0.001; solid grey line), and high fire and high grazing (f: 2, g: 0.06; dotted grey line).

Despite a wealth of empirical research in this field, few quantitative thresholds for dryland degradation have been proposed. This reflects the fact that equilibrium conditions rarely exist in drylands^1^, rendering fixed thresholds meaningless over certain timescales, and that dryland inhabitants regularly change their coping strategies in response to climatic and resource stress^23^. Nevertheless, it can be helpful to determine amount of time a dryland vegetation system spends below specific threshold population densities. Figure 6 categorises the seasonal frequency of different grass and shrub covers during the 21^st^ century at Maun and Tsabong, according to the fire/grazing regime. For example, at Maun (Fig. 6a), a low fire, high grazing scenario results in grass cover dropping below an arbitrary 20% threshold for 98% of the century. In a high fire, high grazing scenario, grass cover drops below the 20% threshold only 4% of the time. At Tsabong (Fig. 6b), a low fire, low grazing scenario is most favourable for high grass cover (grass drops below the 20% threshold 51% of the time, compared with 78–97% of the time in the other three regimes shown). At both Maun (Fig. 6c) and Tsabong (Fig. 6d), a high fire, low grazing scenario results in shrub populations dropping below the 20% threshold most often, at 57% and 95% of the time respectively. In this way, categorising the time spent in sub-threshold conditions allows land managers to better understand the potential medium- to long-term effects of their strategies.

Thomas *et al*.^36^ demonstrated, at the relatively coarse dunefield scale, that the Kalahari Desert could experience significant reactivations as a result of 21^st^ century climate change. The ViSTA model represents an evolution in the reduced complexity approach adopted by Thomas *et al*.^36^, and others before and since, by explicitly including known feedbacks between vegetation growth, wind flow and sediment transport. Crucially, our cellular automaton method allows ecogeomorphic processes to be simulated at appropriately fine spatial and temporal scales. Findings from this study suggest that the impact of climate change alone on the landscape may not be as drastic as predicted by Thomas *et al*.^36^. At Maun, vegetation cover could decrease slightly throughout the century because of declining summer precipitation, which may in turn lead to enhanced sediment transport and land degradation^26, 66^. A late onset and decline in the main rainfall season (DJF), which has been observed in most of southern Africa over recent decades^67, 68^, will have potentially severe implications for maize growing in this region^69^. At Tshane and Tsabong, the existing proximity of the desert system to ecogeomorphic thresholds results in some interannual variability in landscape response to climatic change, but no clear long-term trends. These findings are independent of the RCP used.

However, the inclusion of fire and grazing dynamics in our simulations results in vastly differing potential landscape responses through the 21^st^ century. Fire frequency has a primary impact on vegetation cover, and together with grazing pressure dictates the proportion of grasses and shrubs in the vegetation system. Reduced fire intensity clearly results in more established shrub populations at the expense of grass populations. This in turn affects sediment mobility, potentially enhancing land degradation processes in barren soil patches. Our study therefore suggests that human activities could shape a range of Kalahari landscapes, above and beyond climate change impacts. To protect agricultural and pastoral livelihoods, which generally benefit from high grass cover, it will be necessary to carefully balance fire cycles (both anthropogenic and natural) with animal stocking rates. This is usefully predicted by comparing the potential state of the vegetation system at different thresholds, using density-frequency plots based on several fire/grazing scenarios. If appropriate land management strategies are not implemented in vulnerable dryland regions, the socio-economic and environmental consequences could be significant.

## Methods

### ViSTA model

The Vegetation and Sediment TrAnsport model (ViSTA) is a coupled cellular automaton (CA) model designed to simulate the evolution of semi-vegetated dryland landscapes. The model consists of two coupled modules that interact with each other over various timescales: (i) a vegetation model (adapted from Bailey^6^) that simulates vegetation growth in response to environmental stresses resulting from climate and land use changes, and (ii) a sediment transport model that moves sediment across the model domain according to spatially varying wind velocities. The coupled scheme is formulated such that the distribution of vegetation (Module 1) alters local wind flow characteristics (Module 2a), thus impacting sediment flux patterns over the surface (Module 2b), which in turn affects vegetation growth (Module 1) through ecological feedbacks. The ViSTA model also incorporates several sub-modules that can be activated to simulate herbivore/grazing, fire and rain events, which have a primary impact on the state of the vegetation (Module 1).

All modules in the ViSTA model rely on local neighbourhood operations to produce dynamic responses from basic rules centred on each discrete part of the grid. All grid cells hold a variety of attributes (including sand height, soil moisture and nutrient levels, and vegetation characteristics such as plant type, height and porosity) that are altered by applying transition rules during each timestep. The state of all cells at the end of that timestep becomes the initial state of all cells at the beginning of the next timestep. Transition rules operate on cells based on the local neighbourhood of those cells (a property known as locality), which encodes a form of spatial association into the system dynamics generated by a CA^70^. The transition rules encoded into Modules 2a and 2b (wind and sediment transport) are parameterised as much as possible using empirical, field-based data (see Mayaud *et al*.^39^).

#### Vegetation growth (Module 1)

In the vegetation growth module, an extended-Moore neighbourhood (composed of five concentric shells) is used to calculate local plant-interaction effects^6, 71^. Grid cells accommodate plant biomass that is representative of three vegetation types: grasses, shrubs and trees. Cells are either occupied (alive) or unoccupied (dead). Most plant dependencies are formulated as a function of arbitrary growth units, such that individuals move along a predetermined nonlinear ‘growth pathway’ to provide equivalent changes in biomass. If a plant experiences sub-optimal moisture conditions, it will grow by a fraction of a full growth unit that reflects the available moisture. If precipitation conditions are particularly harsh, plants can lose growth units and move back down their growth pathways (thus losing biomass). The biomass of a plant determines the strength of competition/facilitation that it exerts on its neighbours, as well as its sensitivity to competition/facilitation from surrounding plants. For each cell, the stress from its neighbourhood is combined with six other factors: (i) its response to precipitation; (ii) cell biomass; (iii) cell age; (iv) sediment balance; (v) grazing; and (vi) fire (see Supplementary Information for a full description of each factor). The total compound stress determines a given plant’s likelihood of dying, and thus being replaced by a new plant.

#### Wind dynamics (Module 2a)

Sediment flux in the ViSTA model is calculated as a function of horizontal wind velocity ($u$), and zones of reduced wind velocity in the ViSTA model are formulated as rectangular ‘corridors’ of recovery, following the theoretical approach of Okin^72^. All vegetation elements distributed on the surface are assumed to produce a downwind zone of affected wind velocity. The recovery length of wind velocity downwind of vegetation elements is controlled by element height and porosity^73^. Wind flow recovers exponentially in the wake of grasses and shrubs, and logistically in the wake of trees. Airflow compression is simulated over surface topography.

#### Sediment movement (Module 2b)

Sediment transport dynamics in the ViSTA model are based on Werner’s^74^ algorithm for simulating dunes in arid environments. Volumes of sediment are transported across the model domain based on probabilities of erosion (*p*_*e*_) and deposition (*p*_*d*_) for each cell (depending on sand cover) and the wind velocities derived in Module 2a. Each cell is polled once per iteration (‘polling without replacement’^75^). The total amount of sediment eroded from a given cell is calculated as a function of the cell’s wind velocity, using the semi-empirical flux relationship of Dong *et al*.^76^. For the purposes of simplification, we assume a bulk density of sand of 2000 kg m^−3^ in all our experimental runs, but it should be noted that sand textural characteristics vary locally over the Kalahari region^77^. The eroded sediment is distributed downwind along a deposition pathway specifically calculated for each source cell. Shadow zones (15° to the surface) exist in the lee of topography, where no erosion and complete deposition occur. Avalanching occurs to maintain a critical angle of repose (30° for a bare sand cell, 40° for a vegetated cell).

More details on the ViSTA model are provided in the Supplementary Information, as well as the open-access Supplementary Information of Mayaud *et al*.^39^.

#### Model limitations

Like all models to a certain extent, ViSTA relies on various parameterisations for describing processes that occur within it. Modelled relationships between soil moisture and vegetation growth are currently based on arbitrary growth units linked to observed relationships between precipitation and vegetation state dominance (see Supplementary Information). These parameterisations could be improved using existing datasets on dryland soil moisture distribution^78^, supplemented by local- to regional-scale moisture patterns derived from remote sensing and land-surface modelling^79^. Growth functions linking sediment balance with vegetation growth are currently derived from a combination of qualitative vegetation behaviour described in the literature and trial and error^75^. More empirical, field-based data are needed to improve these sediment balance parameterisations. In its current form, ViSTA simulates human management impacts in simplified probabilistic (e.g. fire) or spatially averaged (e.g. grazing) ways. Future development of ViSTA will involve more sophisticated coupling between the human system and landscape dynamics, for instance using cellular automata of fire front spread^80^ and agent-based modelling of animal foraging and avoidance behaviour^81^.

### Climate projections

Climate data were acquired from the open-source Climate Information Platform^82^. CIP provides future climate projections (until 2100) based on several models from the fifth Coupled Model Intercomparison Project (CMIP5) for RCP 4.5 and RCP 8.5. The projections are downscaled locally to specific weather station locations through the statistical downscaling method of Hewitson & Crane^83^. This method uses modelled circulation fields to generate projections, by associating appropriate weather states based on observations, thus obviating the need for bias correction. A subset of three models was chosen from the CIP ensembles, based on Dieppois *et al*.’s^84^ ranking of CMIP5 model performance over southern Africa: CNRM-CM5, MIROC5 and MRI-CGCM3 (see Supplementary Note 2). For each of the three study sites, precipitation and temperature data were averaged across the three models at a monthly timestep. Additionally, observed climate datasets for each study site were acquired from the Botswana Department of Meteorology, to verify the modelled historical climate data and to indicate the reliability of future model projections (Supplementary Figure 3).

Wind was imposed in Module 2a using wind velocity distributions derived from a 20-year dataset measured at Twee Rivieren in the southwest Kalahari. The distribution of velocity data in each season was assessed separately (see Supplementary Note 2 and Supplementary Figure 4), and a sample was drawn randomly from the appropriate seasonal Weibull distribution at the start of each model iteration. The velocity threshold for entrainment was set at 5.1 m s^−1^, based on realistic potential thresholds measured in the Kalahari^85^.

### Model experiments

For our chosen key years (2030, 2060 and 2090) and the baseline year (2000), we used mean projected precipitation data from the two decades around each year to improve reliability (e.g. data for 2020–2040 were used to represent the aggregate ‘year’ 2030). The sporadic nature of wind events in natural desert environments was simulated by randomly selecting wind velocities from Weibull distributions for each season, based on long-term wind data collected at Twee Rivieren in the southern Kalahari (see Supplementary Note 2). Wind directions were randomly selected from a probability density function based on the direction data presented in Fig. 1.

For the equilibrium experiments, the model was run for each site, at the chosen years and for both RCPs, for the equivalent of 200 model years over a grid of 150 × 150 cells. Low variability (standard deviation, $\sigma$ = 30 mm annual equivalent) and high variability ($\sigma$ = 160 mm annual equivalent) precipitation conditions were simulated based on the variability range in observed historical data. The vegetation module was run every 3 model months, with 20 wind events between each vegetation module update. The domain was randomly initiated with 90% vegetation cover and random sediment depth. Landscape characteristics were calculated as means over the last 10 years of each run, and normalised to those of the baseline year (2000), for RCP 4.5.

For the transient run experiments, the model was initialised with the equilibrium vegetation conditions observed for the year 1960 for each respective site, and forced with 140-year precipitation trajectories from the CIP datasets (i.e. 1960–2100). At each site and for both RCPs, 10 model runs were performed and the resulting landscape characteristics aggregated to indicate the average response. Variability was introduced by randomly sampling from a normal distribution around the projected precipitation trajectory for each run, with a standard deviation ($\sigma$) calculated from 5-year moving averages of the standard deviation for each season. The same grid and module update parameters were used as for the equilibrium experiments.

For the fire and grazing experiments, the model was run in the same way as for the transient run experiments, forced with precipitation trajectories from 1960–2100. Runs were performed for Maun and Tsabong only under RCP 4.5, to capture a range of dynamics in the Kalahari under a stabilising climate. At both sites, three stocking rates (given as large stock units (LSU) per hectare (ha)) were imposed to simulate different grazing pressures typical of Kalahari rangelands^86, 87^: 0.001 LSU ha^−1^ (low grazing), 0.01 LSU ha^−1^ (medium) and 0.06 LSU ha^−1^ (high). Three fire frequencies were also imposed: once every 10 years (low), once every 5 years (medium) and once every 2 years (high). 3 model runs were performed for each scenario, and the resulting aggregate landscape characteristics were calculated as means over the 10 years around each chosen date (e.g. 2025–2035 for 2030).

The model state parameters for all experiments presented in this study are summarised in Supplementary Table 2.

### Data availability

The authors declare that most of the data supporting the findings of this study are available within the paper, and its Supplementary Information files. Projected climate data were acquired from the open-access Climate Information Platform (http://cip.csag.uct.ac.za/webclient2/datasets/africa-merged-cmip5/). Any other data supporting the findings are available from the corresponding author upon request.

### Code availability

The model is implemented in the Python® programming language (Python 3.5.0 64bits, Qt 4.8.7, PyQt4 (API v2) 4.11.4 on Darwin; Spyder® development environment), using code written by the authors. A full version of the ViSTA model code is freely available on GitHub (https://github.com/jeromemayaud/ViSTA).

## Electronic supplementary material

Supplementary Information: Modelled responses of the Kalahari Desert to 21st century climate and land use change
