# Supplementary material for: Modelled responses of the Kalahari Desert to 21st century climate and land use change
Source: Sci Rep. 2017 Jun 20;7:3887. doi: 10.1038/s41598-017-04341-0 (PMC5478674; doi:10.1038/s41598-017-04341-0)
Supplement: Supplementary file 1 — Supplementary Information: Modelled responses of the Kalahari Desert to 21st century climate and land use change [file 41598_2017_4341_MOESM1_ESM.pdf]

Supplementary Information:

Modelled responses of the Kalahari Desert to 21st century climate and land use change

Jerome R. Mayaud[1], Richard M. Bailey[1], Giles, F. S. Wiggs [1]

[1]School of Geography and the Environment, Oxford University Centre for the Environment, University of Oxford, Oxford, OX1 3QY, UK

NOTES

1. ViSTA cellular automaton model

The Vegetation and Sediment TrAnsport (ViSTA) model consists of two interacting coupled modules: (i) a vegetation model (Module 1) that simulates vegetation growth, and (ii) a sediment transport model that moves sediment across the model domain according to spatially varying wind speeds (Module 2).

1.1. Vegetation growth (Module 1)

The vegetation module consists of an adapted version of Bailey's (2011) CA model for simulating semi-arid vegetation. Seven factors govern a cell's probability of survival (if it is vegetated) or probability of being colonised by a plant: (i) neighbourhood effects; (ii) response to precipitation; (iii) cell biomass; (iv) cell age; (v) sediment balance (i.e. plant response to sediment erosion/deposition); (vi) grazing; and (vii) fire. Most plant dependencies in the model are formulated as a function of arbitrary growth units; these dependencies are parameterised using empirical data where available. Biomass (B) controls the strength of competitive and facilitative interactions between neighbouring plants. Individual plants move along nonlinear 'growth pathways' more or less rapidly, depending on the harshness of the surrounding growth conditions.

The neighbourhood of a given cell is defined by five concentric shells in an extended-Moore neighbourhood (i.e. a two-dimensional lattice composed of a central cell and the 120 cells that surround it). Each shell is assigned a competition coefficient (c_i), with positive values representing net facilitative effects at close distance (shells 1 and 2), negative values representing net competitive effects (shells 4 and 5), and zero being neutral (shell 3). Changing the grid cell size within a realistic range (10^{-1}–10^{1} m) does not significantly affect shell interactions and thus model behaviour (see also Mayaud et al., 2017). The modelled facilitation strength grows monotonically with plant biomass (B), from zero to a maximum at full biomass maturity (B_{max}), and remains constant thereafter. Modelled competition rises monotonically with biomass until B_{max}, and then reduces as biomass increases past B_{max}. Smaller plants in the model benefit more from facilitation than larger plants. Cells are at their most resilient and sensitivity to competition reaches a minimum when plants reach B_{max}; beyond this, sensitivity to competition rises again.

The biomass-dependent contributions from all the occupied cells in a given cell's neighbourhood are summed and combined with stress from incoming precipitation, (ρ, mm), to give a total neighbourhood score (T) at each time-step:

$$T = \sum_{i=1}^{2}\left(\sum_{j=1}^{i\times 8}\left(a_{i,j}c_i k_{f(i,j)}\right)\right)s_f + \sum_{i=3}^{5}\left(\sum_{j=1}^{i\times 8}\left(a_{i,j}c_i k_{c(i,j)}\right)\right)s_c + \rho_S$$

Equation 1

where $a_{i,j}$ is the value of the jth cell in the ith shell (a = 1 for an occupied (live) cell and a = 0 for an unoccupied cell); c_i is the competition/facilitation coefficient of shell i (i = 1…5); k_c is the competition strength (< 0); k_f is the facilitation strength (> 0); s_c is the sensitivity to competition (≥ 0); s_f is the sensitivity to facilitation (> 0); and ρ_S is the precipitation stress imposed globally on the system (see Equation 1). In the case of k_c, k_f, s_c and s_f, whose values depend on plant type, the form of the normal distribution is used in the definition of the biomass-dependence. The function $N(B)$ describes a standard normal distribution:

$$\boldsymbol{N(B)} = \frac{\mathbf{1}}{\boldsymbol{w\sqrt{2\pi}}} \boldsymbol{e}^{-\frac{(\boldsymbol{B}-\boldsymbol{p})^2}{2\boldsymbol{w}^2}}$$

Equation 2

The function $N(B)$ forms the basis for k_c, k_f and s_c:

$$k_c(B) = \begin{cases} 60N(B), & (grass) \\ 240N(B), & (shrub) \\ 380N(B), & (tree) \end{cases}$$

Equation 3

$$k_f(\gamma) = \begin{cases} 60N(\gamma), & (grass) \\ 240N(\gamma), & (shrub) \\ 3800N(\gamma), & (tree) \end{cases}$$

$$\gamma = \begin{cases} B, & B \leq B_{max} \\ B_{max}, & B > B_{max} \end{cases}$$

Equation 4

$$s_c(B) = \begin{cases} 1 - 54N(B), & (grass) \\ 1 - 216N(B), & (shrub) \\ 1 - 342N(B), & (tree) \end{cases}$$

Equation 5

$$s_f(B) = \begin{cases} 1 + 2e^{-0.208\dot{3}B}, & (grass) \\ 1 + 2e^{-0.041\dot{6}B}, & (shrub) \\ 1 + 2e^{-0.02604B}, & (tree) \end{cases}$$

Equation 6

where p and w are the peak position and width parameter respectively in B, and differ according to plant type (grasses: p = 60, w = 24; shrubs: p = 300, w = 96; trees: p = 480, w = 152).

The precipitation stress (ρ_S) is defined as:

$$\rho_S = m.\left(\frac{\rho - \rho_{mid}}{\rho_{mid}}\right)$$

Equation 7

where ρ_{mid} represents the precipitation level that has neither an added beneficial nor detrimental effect on a plant, and m is a scaling parameter. Different precipitation stress relationships are assumed for grasses, shrubs and trees (for grasses, ρ_{mid} = 180 mm yr^{-1} and m = 1; for shrubs, ρ_{mid} = 200 mm yr^{-1} and m = 1; and for trees, ρ_{mid} = 500 mm yr^{-1} and m = 0.8).

The T score is normalised to the range 0–1 (asymptotic at very high and very low values of ρ_S), giving the normalised adjacency score, $\tilde{T}$:

$$\tilde{T} = \frac{1}{1 + e^{-5T}}$$

Equation 8

A precipitation response lag (Wand, 1999) is introduced by using $\tilde{T}$ to determine the proportion of a theoretical growth unit by which all plants can grow over a given iteration. A monthly growth gain unit (G_{unit}) increases monotonically from -0.05 (at $\tilde{T}$ = 0) to +1 (at $\tilde{T}$ = 1):

$$G_{unit} = \frac{1.05}{1 + e^{-k_1(\tilde{T} - \tilde{T}_{mid})}} - 0.05$$

Equation 9

where the k_1 parameter determines the steepness of the curve (k_1 = 15 in the present model) and $\tilde{T}_{mid}$ is the value of the sigmoid's midpoint ($\tilde{T}_{mid}$ = 0.4 in the present model). In this way, G_{unit} can in some instances be negative, reflecting a plant losing biomass in particularly harsh conditions.

The cumulative growth (G_{cum}) of a plant, which is used to determine the actual biomass (B) this represents along its growth pathway, is calculated as:

$$G_{cum} = \sum_{i=1}^{iter} G_{unit}$$

Equation 10

If the vegetated cell dies, its cumulated growth returns to zero. The growth pathway is formulated as a logistic curve (cf. Erickson, 1976):

$$B = \frac{B_{max}}{1 + e^{-k_2(G_{cum} - G_{mid})}}$$

Equation 11

where B_{max} is the maximum biomass a plant can achieve over its lifetime (equivalent to full age maturity in months), G_{mid} is the value of the sigmoid's midpoint, and the k_2 parameter determines the steepness of the curve (grasses: G_{mid} = 25; k_2 = 0.14; shrubs: G_{mid} = 130; k_2 = 0.025; trees: G_{mid} = 210; k_2 = 0.016).

A plant's biomass B is converted into an equivalent vegetation height (h):

$$h = h_{max}\left(\frac{B}{B_{max}}\right)$$

Equation 12

where h_{max} is the maximum height (m) to which a plant can grow. The resultant value of h for each grid cell is exported to Modules 2a and 2b to determine wind speed patterns and sediment transport rates across the model domain.

For each cell, the stress from its neighbourhood and from precipitation is combined with other stresses (due to accumulated drought (D_S), due to age (Z_S), due to sedimentation balance (Sed_S), due to grazing (G_S), and due to fire (F_S)), which themselves depend on plant type. These various stresses are described below. The total compound stress determines a given plant's likelihood of dying, and thus being replaced by a new plant.

When $\tilde{T}$ < 0.5 (Equation 8), a given plant experiences a period of generally unfavourable conditions, which for the purposes of this model is termed a 'plant drought'. The severity of a plant drought is proportional to the number of consecutive (unbroken) months where $\tilde{T}$ is lower than 0.5, and is a function of plant type and the magnitude of $\tilde{T}$. The consecutive number of plant droughts (D_{con}) is given by:

$$D_{con} = \begin{cases} \sum \frac{0.5 - \tilde{T}}{0.5}, & \tilde{T} < 0.5 \\ 0 & , \tilde{T} \geq 0.5 \end{cases}$$

Equation 13

The stress that a plant experiences due to drought (D_S) is an exponential function of D_{con}:

$$D_S = 1 - e^{-bD_{con}}$$

Equation 14

where b controls the saturation rate of the exponential and is dependent on plant biomass.

Stress due to age (Z_S) is given as:

$$Z_S(z) = \begin{cases} \begin{cases} \frac{0.2 - 12N(z)}{5}, & z \leq 60 \\ \frac{1 - 120N(z)}{5}, & z > 60 \end{cases} & (grass) \\ \begin{cases} \frac{0.2 - 48N(z)}{5}, & z \leq 300 \\ \frac{1 - 240N(z)}{5}, & z > 300 \end{cases} & (shrub) \\ \begin{cases} \frac{0.2 - 76N(z)}{5}, & z \leq 480 \\ \frac{1 - 480N(z)}{5}, & z > 480 \end{cases} & (tree) \end{cases}$$

Equation 15

where z = cell age and $z \geq 1$ for occupied cells.

The sediment-balance stress (Sed_S) is calculated as a function of sedimentation balance (annual equivalent, in m) for each vegetated grid cell. These are partly based on the elementary growth functions presented by Nield and Baas (2008). There is much debate as to the most appropriate sediment-balance/growth curves for ecogeomorphic modelling, partly because relevant ecological studies tend to focus on detailed physiological measurements that may not be useful for geomorphology (Barchyn & Hugenholtz, 2015). For instance, contrary to Nield and Baas (2008),

models by Duran and Hermann (2006) and Barchyn & Hugenholtz (2012) do not include growth stimulation in their sedimentation response curves. Barchyn & Hugenholtz (2015) define a 'peak vegetation deposition tolerance' in dune environments, which could theoretically be quantified at large enough scales using remote sensing. However, values for this deposition tolerance are currently still lacking for our study region. We therefore chose to employ the well-tested curves of Nield and Baas (2008). In this scheme, grass species experience peak positive Sed_S (i.e. reduced probability of death) in conditions of net deposition. Neutral or negative balances lead to negative Sed_S (i.e. increased probability of death) for grasses, due to the impact of parasites and soil pathogens (Maun, 1998). Invasive woody shrub species prefer less active landscapes than grasses, so Sed_S rapidly declines when significant erosion or deposition occurs. There is relatively little literature on the impact of sedimentation balance on tree growth in drylands, but evidence suggests that some sediment accumulation can be beneficial to tree development (e.g. Wagner et al., 2013). The growth functions are shown in Figure 1.

The stress due to grazing (G_S) is calculated as a function of the stocking rate on the landscape. Since livestock only graze on grasses, but can trample other plants, G_S is always set to 0.01 for both shrubs and trees. For cells colonised by grasses, G_S is given as:

$$G_S = \begin{cases} n\Omega, & \Omega \leq 0.06 \\ 0.90, & \Omega > 0.06 \end{cases}$$

Equation 16

where Ω is the stocking rate (LSU ha^{-1}) and n is the slope factor (n = 15 in the present model).

The stress that a plant experiences due to fire (F_S) is constant for grasses of all ages, due to the high propensity for grassy material to burn at all ages (Danin, 1996), whereas it is calculated as an exponential function of cell age (z) in the case of shrubs and trees:

$$F_S = \begin{cases} 0.66, & (grass) \\ 0.6\left(e^{-lz}\right) & (shrub) \\ 0.4\left(e^{-lz}\right) & (tree) \end{cases}$$

Equation 17

where l controls the saturation rate of the exponential (l = 0.01 in the present model). In this way, trees of a given age are more likely to survive a fire than shrubs of the same age, as is often the case in the Kalahari (Bhattachan et al., 2014; Dougill et al., 2016).

Together, all the stresses experienced by a given cell contribute to its total stress (T_S), which determines to probability the cell dies if it is vegetated:

$$T_S = Sed_S - Z_S(z) - D_S - F_S - G_S$$

Equation 18

T_S serves as a critical value against which a uniformly distributed random value (β) is compared. At each timestep, β is recalculated for each cell. If $\beta \leq T_S$, a dead/empty cell becomes occupied ($a: 0 \rightarrow 1$) and occupied cells live on (a = 1), increasing their age by the appropriate time unit; if $\beta > T_S$, a live cell dies ($a: 1 \rightarrow 0$) and an empty cell remains empty (a = 0).

The recolonisation of an empty cell by a particular vegetation type (grass, shrub or tree) is determined dynamically, such that the proportion of each vegetation type occupying the domain at the end of a given iteration determines the probabilities that the vegetation types recolonise a bare cell at the next iteration:

$$p_{RG} = \frac{(P_G + \alpha_G)}{(1 + \alpha_G + \alpha_S + \alpha_T)}$$

Equation 19

$$p_{RS} = \frac{(P_S + \alpha_S)}{(1 + \alpha_G + \alpha_S + \alpha_T)}$$

Equation 20

$$p_{RT} = \frac{(P_T + \alpha_T)}{(1 + \alpha_G + \alpha_S + \alpha_T)}$$

Equation 21

where p_{RG}, p_{RT} and p_{RS} are the probabilities of recolonisation of an empty cell by a grass, tree or shrub, respectively; P_G, P_S and P_T are the current proportions of grass, shrub and tree cells (respectively) in the entire model domain; and α_G, α_S and α_T are

multiplicative factors that act to increase or decrease the recolonisation probabilities of a grass, shrub or tree, respectively. α_G, α_S and α_T are formulated as a function of the precipitation regime (see Mayaud et al., 2017).

1.2. Wind dynamics (Module 2a)

An unobstructed wind velocity (u_{ref}) is derived at each timestep, which represents the mean flow over a flat, sandy surface with no elements. The stochastic nature of airflow (e.g. Rice et al., 1999; Böhner et al., 2003; Klose and Shao, 2012) is introduced by approximating a Gaussian frequency distribution ($\mu = u_{ref}$, $\sigma = 0.1$), from which a wind velocity is randomly chosen for each cell in the domain. This wind velocity is further adjusted depending on vegetation morphology and surface topography.

Species type (here grass, shrub, tree) determines whether an element acts to increase or decrease wind velocity in the wake. For grasses and shrubs, the surface wind velocity in the wake (u_{surf}) is lower than in the absence of plants (u_{ref}), and exponentially recovers with increasing downwind distance:

$$u_{surf} = (u_{ref} - u_0).(1 - e^{-b\frac{x}{h}}) + u_0$$

Equation 22

where u_0 is the minimum wind velocity in the direct lee of the nearest upwind element, $\frac{x}{h}$ is the downwind distance from the nearest element in terms of element height, and b is a fitted coefficient. The coefficient values are based on the parameterisation of Mayaud et al. (2016b):

$$u_0 = u_{ref}(0.0146\theta_{cum} - 0.4076)$$

Equation 23

$$b = 0.0105\theta_{cum} + 0.1627$$

Equation 24

In the case of trees, wind flow in the lee is parameterised using a logistic curve:

$$u_{surf} = 0.4e^{-\frac{(h_t-\mu)^2}{1.13}}\left(1 - \frac{1}{1 + e^{-2\left(\frac{x}{h}-4.2\right)}}\right) + 1$$

Equation 25

To reflect airflow compression over topography, a speedup/slowdown effect is introduced by using an additive factor that scales linearly with the length of a given wind path along a slope. The slope-corrected wind velocity (u_{slope}) for a particular cell is dependent on the wind velocity of the upwind cell ($u_{surf[k-1]}$):

$$u_{slope} = u_{surf[k-1]}.\gamma$$

$$\gamma = \begin{cases} (s_{h[k]} - s_{h[k-1]}).\,\omega, & (s_{h[k]} - s_{h[k-1]}) \neq 0 \\ 1 \quad\quad\quad\quad\quad\quad\quad\quad, & (s_{h[k]} - s_{h[k-1]}) = 0 \end{cases}$$

Equation 26

where $s_{h[k]}$ is the surface height of the polled cell, $s_{h[k-1]}$ is the surface height of the upwind cell, and ω is a change coefficient (set at 0.1 in the current model). In order to prevent runaway wind velocities, the maximum amount of speedup is limited to 100% of the unobstructed wind velocity.

Continuous wind and transport directions are imposed onto the domain using an algorithm described in detail in Mayaud et al. (2017).

1.3. Sediment movement (Module 2b)

At each iteration of Module 2B, a function is applied to determine how much sediment is eroded from each cell. The total amount of sediment eroded from a given cell is calculated deterministically as a function of the cell's wind velocity, using the semi-empirical flux relationship of Dong et al. (2003):

$$Q = a\left(1 - \frac{u_t}{u}\right)^2 \left(\frac{\rho}{g}\right) u^3$$

Equation 27

where Q is the predicted mass flux for the given time interval (kg m^{-1} s^{-1}), u is the mean horizontal wind velocity over the given time interval (m s^{-1}), u_t is the horizontal wind velocity threshold (m s^{-1}), ρ is the density of air (1.25 kg m^{-3}), g is acceleration due to gravity (9.81 m s^{-2}), and a is an empirically fitted constant (a = 0.002, based on the field data of Mayaud et al., 2016).

The flux is converted into an equivalent volumetric flux (m^2 s^{-1}), assuming a bulk density for sand of 2000 kg m^{-3} (e.g. Bailey and Thomas, 2014), and from this the height of the moving sand volume for a given cell width can be derived. The p_e value of a polled cell determines the proportion of the sand volume that is actually eroded from it; p_e varies from 0 to 1 depending on the moisture level of the sediment in the cell and its location relative to shadow zones. The final height of the sand volume is removed from the polled cell and distributed downwind along a deposition pathway specifically calculated for each source cell.

Transport and deposition in the model occurs stochastically, with each cell being characterised by a different p_d value. Destination cells already populated with sediment are assigned a p_d value of 0.7, whilst bare destination cells are assigned a lower p_d value of 0.4. If a cell is vegetated, the p_d value increases proportionally to the plant porosity. The total volume eroded from a source cell is divided according to the p_d of each downwind cell (until a set limit of downwind cells is reached), as well as the length of the wind path traversing each downwind cell. Shadow zones exist in the lee of topography, such that no erosion (p_e = 0) and complete deposition (p_d = 1) occur in regions within an angle of 15° to the horizontal surface.

Avalanching occurs in the direction of steepest descent (regardless of vegetation occupation status) to enforce an angle of repose, induced by gravity. The angle at which avalanching is triggered from the destination cell changes according to the vegetation occupation of the cell (30° for a bare sand cell, 40° for a vegetated cell). Cells are polled randomly, so as to prevent artefacts propagating across the domain as a result of a specific avalanching order.

1.4. Model state parameters

The model state parameters for each experiment presented in this study are summarised in Table 1.

2. Climate data

2.1. Future climate projections

Projections of changes in the climate system in the IPCC framework are made using a hierarchy of climate models (IPCC, 2013). A set of scenarios, termed 'representative concentration pathways' (RCPs), are identified by their approximate total radiative forcing in the year 2100 relative to 1750 (IPCC, 2013). Commonly used scenarios in environmental modelling studies are RCP 4.5, representing stabilisation of greenhouse gas (GHG) emissions, and RCP 8.5, a high GHG emission scenario. Therefore, the RCP 4.5 and RCP 8.5 scenarios were chosen for this study.

There is much debate in the literature concerning how best to combine information from different models, which models to include, and the statistics that should be presented (Frame et al., 2007; Knutti, 2010; McSweeney & Jones, 2013). However, it is generally held that using a range of model projections instead of relying on any single model improves the robustness of analysis (Thornton et al., 2011; James & Washington, 2012; Whetton et al., 2012). In this study, a subset of three models was chosen from the CIP ensembles, based on Dieppois et al.'s (2015) ranking of CMIP5 model performance over southern Africa: CNRM-CM5, MIROC5 and MRI-CGCM3. For each of the three study sites, precipitation and temperature data were averaged across the three models at a monthly timestep.

Figure 2 displays projected changes in annual precipitation over the period 2017–2100, for all three sites under RCP 4.5 and RCP 8.5.

2.2. Verifying climate projections

Observed climate data can be used to verify modelled historical climate, which in turn can inform the reliability of model projections into the future. Figure 3 displays modelled and observed precipitation and temperature data (on a monthly timescale) for Maun, Tshane and Tsabong over historical periods for which observed data were available from the Botswana Department of Meteorological Services. Whilst temperature patterns at the three locations were relatively well back-cast (Figure 3d,e,f), with strong coefficients of determination between observed and modelled data (R^2 = 0.70–0.84; Figure 3h,j,l), the variability in precipitation data was not so well simulated (Figure 3a,b,c), with weak coefficients of determination between observed and modelled data (R^2 = 0.11–0.20; Figure 3g,i,k). The relatively poor representation of past precipitation is a common issue in climate modelling due to large uncertainties in the underlying forcing factors (Raisanen, 2007). The ability of the chosen models to faithfully replicate past temperature changes provides some indication of reliable model performance. The uncertainty in precipitation changes is accounted for in our experimental runs by imposing random precipitation variability, based on the variability characterising the two decades around each chosen year (i.e. 2020–2040, 2050–2070 and 2080–2100).

2.3. Wind forcing

Hourly wind velocity data were acquired for Twee Rivieren in the southwest Kalahari, based on the availability of long-term wind data available from the Botswana Meteorological Service. Wind velocity data at 08:00 (morning), 14:00 (afternoon) and 20:00 (evening) were selected for each day between 1st January 1994 and 31st December 2013. These times were chosen to account for diurnal wind variations. Days with missing data points were removed, and the resulting data were grouped by season (MAM, JJA, SON and DJF). The distribution of the data in each season was assessed using MATLAB's built-in 'wblplot' function, which graphically assesses whether the data could come from a Weibull distribution (Figure 4). For each month grouping, the maximum likelihood estimates of the 2-parameter Weibull distribution were calculated using MATLAB's built-in 'wblfit' function. These are summarised in Table 2. Weibull distributions of wind velocity were generated using the parameters

for each month grouping, and samples were drawn randomly from the appropriate distribution at each model iteration.

3. Vegetation transects

8 vegetation transects were conducted by R. Bailey & G. Wiggs in September 2010 to characterise vegetation cover within 150 km of the three study sites (2 transects at Maun, 3 transects at Tshane and 3 transects at Tsabong). The vegetation transects were 50 m in length and 1 m wide; along each transect the height, basal width and canopy width of every plant was recorded at a centimetre-scale resolution. Plants were categorised as either herbs and grasses, shrubs or trees. Vegetation proportions were calculated by dividing the canopy width for each plant type by the total canopy width. Vegetation proportions were then averaged across the measurement transects for each site.

4. Oral history of shrub encroachment

In addition to climate model datasets, oral history data were collected on a farm located in the southwest Kalahari Desert, just north of the Auob River. The farm extends across approximately 13,000 h.a. of vegetated linear dunes, with a variety of dryland grasses, shrubs and trees. The sole current farmer of the property was interviewed informally over a period of several weeks in the period August–October 2014 and in September 2016.

In the 1980s, the farmer recalls a landscape much more populated by grasses than shrubs. Whilst wild game moved through in large numbers, placing pressure on the vegetation system, their manure and urine helped to fertilise the land and contributed to rapid grass recolonisation. The lack of human management of wildfires (which are mainly caused by lightning in this region) also helped to maintain low shrub populations. From the 1990s onwards, the region experienced increased agricultural intensification and fence construction for wildfire control. This led to greater grazing pressures and severe shrub encroachment in some areas. Whereas most dunes were covered with grasses ~40 years ago, according to the farmer, much of the

heavily grazed farmland now consists of bare, intermittently active dune crests. The region's strong northeasterly winds result in a relatively slow southwesterly migration of the most denuded dunes.

In an attempt to reverse shrub encroachment on his land, the farmer started using herbicide that targets shrubs only (known as 'de-bushing'; Reed et al., 2015), and allows grasses to dominate the system within 2–3 years. On this particular farm, the application of herbicide in late 2009 resulted in the widespread replacement of *Rhigozum trichotomum* woody shrubs (Figure 5a) with perennial grasses such as *Stipagrostis amabilis* and *Stipagrostis uniplumis* (Figure 5b,c).

5. Impact of precipitation variability

Bailey (2011) showed that conditions of fluctuating generic stress can result in readjustments of vegetation patterns over time, and that the magnitude of stress variability can impact post-perturbation recovery in semi-arid systems. It is therefore important to investigate the impact of precipitation variability on landscape response within the fully-coupled ViSTA model. Variability was imposed by randomly sampling precipitation from a normal distribution around a series of idealized precipitation scenarios: no trend (Figure 6), v-shaped trend (Figure 6), decreasing trend (Figure 7), and increasing trend (Figure 8). For each scenario, no variability, low variability (standard deviation, σ = 50 mm annual equivalent) and high variability (σ = 100 mm annual equivalent) conditions were imposed, based on realistic variabilities observed in historical data at our study sites.

In the no-trend scenario (Figure 6), a lack of variability resulted in a baseline 'equilibrium' state of high, non-patterned vegetation cover, with stable proportions of grass, shrubs and trees. The addition of variability led to pattern formation, with more dramatic population declines at high variability, and grass:shrub ratios that fluctuated significantly. In the v-shaped trend scenario (Figure 6), distinct labyrinthine patterns emerged as vegetation cover declined in response to decreasing precipitation. Variability in the precipitation signal resulted in a less rapid, predictable decline than the no-variability case, because occasional peaks in precipitation helped to rapidly repopulate existing vegetation patches. In the decreasing trend scenario (Figure 7), greater variability led to an earlier decline in population density, as well as an earlier

appearance of patterning (by up to ~60 years) At low average precipitation levels, occasional peaks result in grasses and shrubs to grow rapidly (occasionally to cover of 60–90%), but these die off quickly in subsequent low-precipitation years. Conversely, in the increasing trend scenario (Figure 8), the presence of variability allowed grasses to establish quickly in response to vegetation peaks and, eventually, to form viable patches earlier than in the no-variability case. An intermediate level of variability provided the best conditions for vegetation regrowth, possibly because high variability leads to occasional precipitation lows that drive the vegetation system back down its recovery trajectory. Greater fluctuations in grass:shrub ratios in the high variability case suggest that the system may be closer to flipping to a shrub-encroached landscape.

6. Equilibrium conditions

In addition to the equilibrium landscape characteristics presented in the main manuscript, Figure 9 displays normalised plant ages at the chosen years across all three sites. At Maun, for both variability scenarios and both RCPs, grasses and shrubs on average live increasingly longer until 2060, then experience a decline in age at 2090. Trees tend to live longer as the century progresses. At Tshane, in most cases grasses and shrubs tend to live longer throughout the century than in 2000, particularly in the RCP 8.5 scenario. Average ages for trees do not tend to vary as strongly. At Tsabong, a clear decreasing trend in average ages for grasses and shrubs is evident for RCP 8.5, but for RCP 4.5 high variability leads to an increase in average ages in 2090. Trees do not experience a significant change in average age.

The strength of the feedbacks between sediment movement and vegetation growth can be modified depending on the plant types and environments. Figure 10 shows the effect of varying the ‘sediment importance factor’ in the ViSTA model. This multiplicative factor changes the weighting of the sediment stress (Sed_S) in the formulation for the total stress (T_S) (see Equation 18). The sediment importance factor is at least greater than zero (i.e. the movement of sediment must have at least some impact on vegetation growth), but its upper bound is poorly constrained by a lack of appropriate experimental data. The sediment importance factor affects the population density (Figure 10a), average sediment transport (Figure 10b) and

grass:shrub ratios (Figure 10c) at equilibrium. These landscape characteristics do not change linearly with the value of the factor, demonstrating the potential existence of several equilibria that depend on sediment feedback strength.

Figure 11 shows results from two additional equilibrium experiments. Summer (DJF) rainfall was varied between 80–120 mm yr^{-1} to verify the impact on grass:shrub ratio and population density in equilibrium state (Figure 11a). As summer rainfall increases, population density increases whilst grass:shrub ratios decline (i.e. greater shrub encroachment). This occurs because of more favourable conditions for shrub establishment over grasses. The effect of varying fixed shrub porosities on total sediment transport is shown in Figure 11b. Although there is significant seasonal variability in transported sediment due to seasonal vegetation cover, sediment transport is greater when the model is populated with high-porosity (90%) shrubs compared with low-porosity (10%) shrubs. A landscape composed of intermediately porous shrubs (50%) experiences the lowest sediment transport rates, reflecting findings that intermediately porous elements strike a compromise between the strength of their sheltering effect and the downwind distance over which it is effective (Musick et al., 1996; Lee et al., 2002; Mayaud et al., 2016c).

7. Additional references

Bailey, R.M. & Thomas, D. S. G. A quantitative approach to understanding dated dune stratigraphies. *Earth Surf. Proc. Land,* **39**, 614–631 (2014).

Barchyn, T.E. & Hugenholtz, C.H. Aeolian dune field geomorphology modulates the stabilization rate imposed by climate. *J. Geophys. Res. Earth Surf.,* **117**, F02035 (2012).

Barchyn, T.E. & Hugenholtz, C.H. Predictibility of dune activity in real dune fields under unidirectional wind regimes. *J. Geophys. Res. Earth Surf.,* **120**, 159–182 (2015).

Bhattachan, A., D'Odorico, P., Dintwe, K., Okin, G.S. & Collins, S.L. Resilience and recovery potential of duneland vegetation in the southern Kalahari. *Ecosphere*, **5**, 1–14 (2014).

Böhner, J., Schäfer, W., Conrad, O., Gross, J. & Ringeler, A. The WEELS model: Methods, results and limitations. *Catena*, **52,** 289–308 (2003).

Danin, A. *Plants of Desert Dunes,* Springer Verlag, Berlin (1996).

Duran, O. & Hermann, H.J. Vegetation against dune mobility. *Phys. Rev. Lett.,* **92**, 188001 (2006).

Erickson, R.O. Modeling of plant growth. *Ann. Rev. Plant. Phys.,* **27**, 407–434 (1976).

Frame, D.J. et al. Probabilistic climate forecasts and inductive problems. *Phil. Trans. Royal. Soc. A.,* **365**, 1971–1992 (2007).

Klose, M. & Shao, Y. Stochastic parameterization of dust emission and application to convective atmospheric conditions. *Atmos. Chem. Phys.*, **12**, 7309–7320 (2012).

Knutti, R. The end of model democracy? *Clim. Change,* **102**, 395–404 (2010).

Mayaud, J.R., Bailey, R.M., Wiggs, G.F.S. & Weaver, C.M. Modelling aeolian sand transport using a dynamic mass balancing approach. *Geomorphology,* **280,** 108–121 (2016).

McSweeney, C.F. & Jones, R.G. No consensus on consensus: the challenge of finding a universal approach to measuring and mapping ensemble consistency in GCM projections. *Clim. Change,* **119**, 617–629 (2013).

Nield, J.M. & Baas, A.C.W. Investigating parabolic and nebkha dune formation using a cellular automaton modelling approach. *Earth Surf. Proc. Land.*, **33**, 724–740 (2008).

Raisanen, J. How reliable are climate models? *Tellus,* **59***,* 2–29 (2007).

Rice, M.A., McEwan, I.K. & Mullins, C.E. A conceptual model of wind erosion of soil surfaces by saltating particles. *Earth Surf. Proc. Land.*, **24,** 383–392 (1999).

Wand, S.J.E. A preliminary study of the responsiveness to seasonal atmospheric and rainfall patterns of wash woodland species in the arid Richtersveld. *Plant Ecol., 142,* 149–160 (1999).

Wagner, B., de Leeuw, J., Njenga, M., Iiyama, M. & Jamnadass, R. *Towards Greater Resilience in the Drylands: Trees Are the Key*, Nairobi, Kenya (2013).

Whetton, P. et al. Use of Representative Climate Futures in impact and adaptation assessment. *Clim. Change,* **115**, 433–442 (2012).

FIGURES

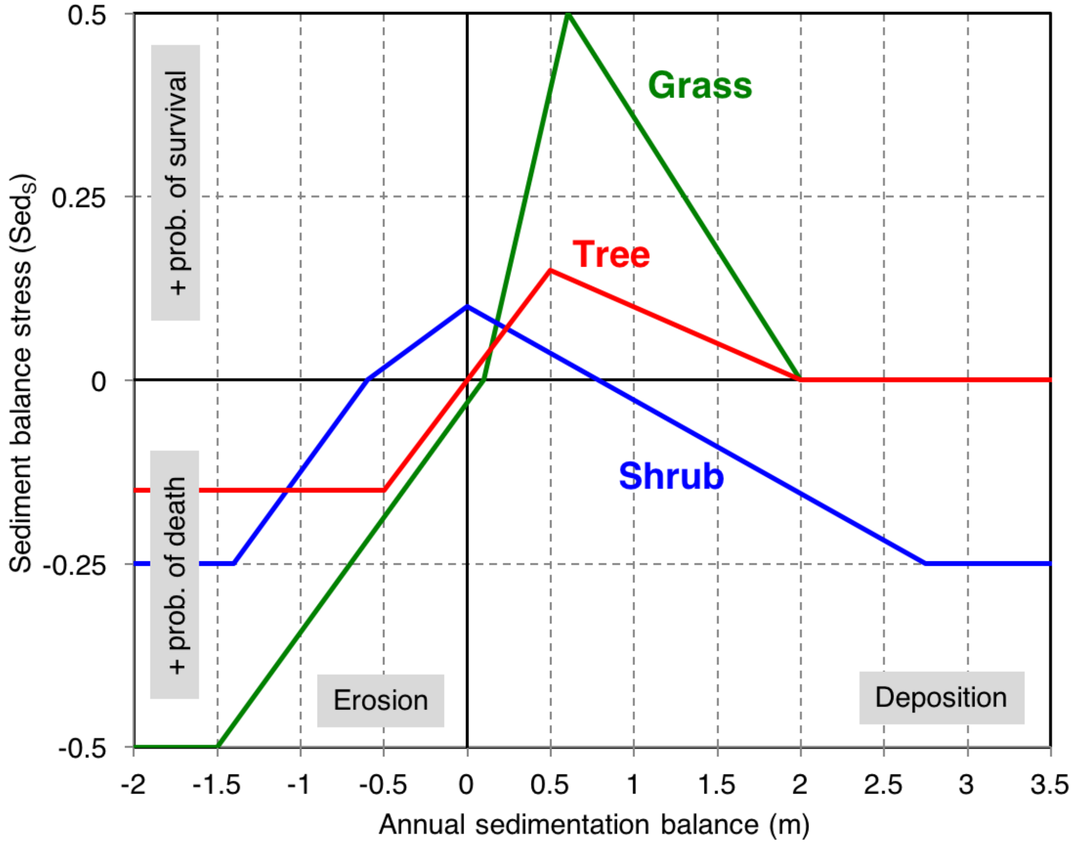

Figure 1 Functions for stress due to sedimentation balance (Sed_S), representing the annual response to burial and/or erosion conditions for the different vegetation types (adapted from Nield and Baas, 2008a, p.731).

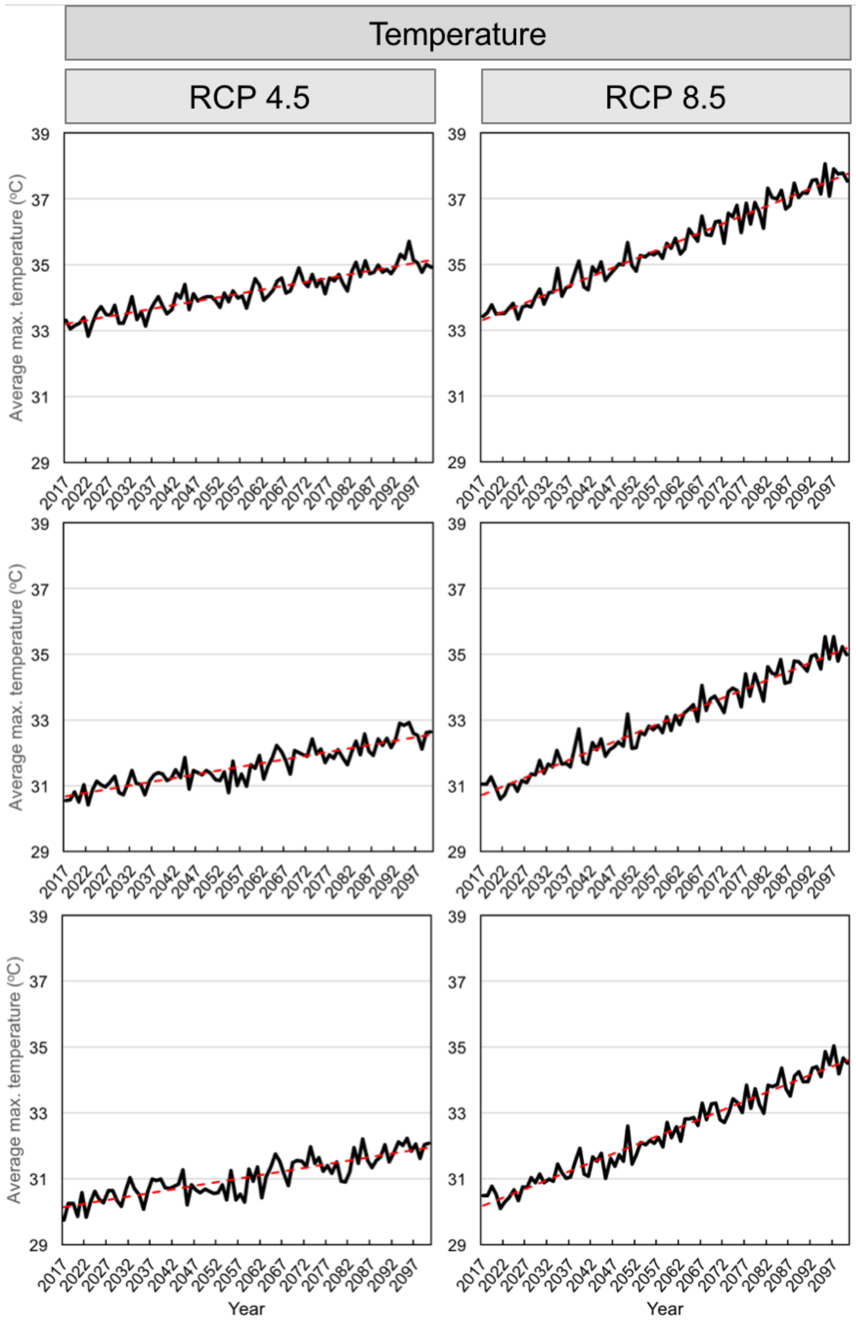

Figure 2 Projected changes in average maximum daily temperature over the period 2017–2100, for Maun, Tshane and Tsabong, under the RCP 4.5 and RCP 8.5 scenarios. Dotted red lines show linear best fit trends.

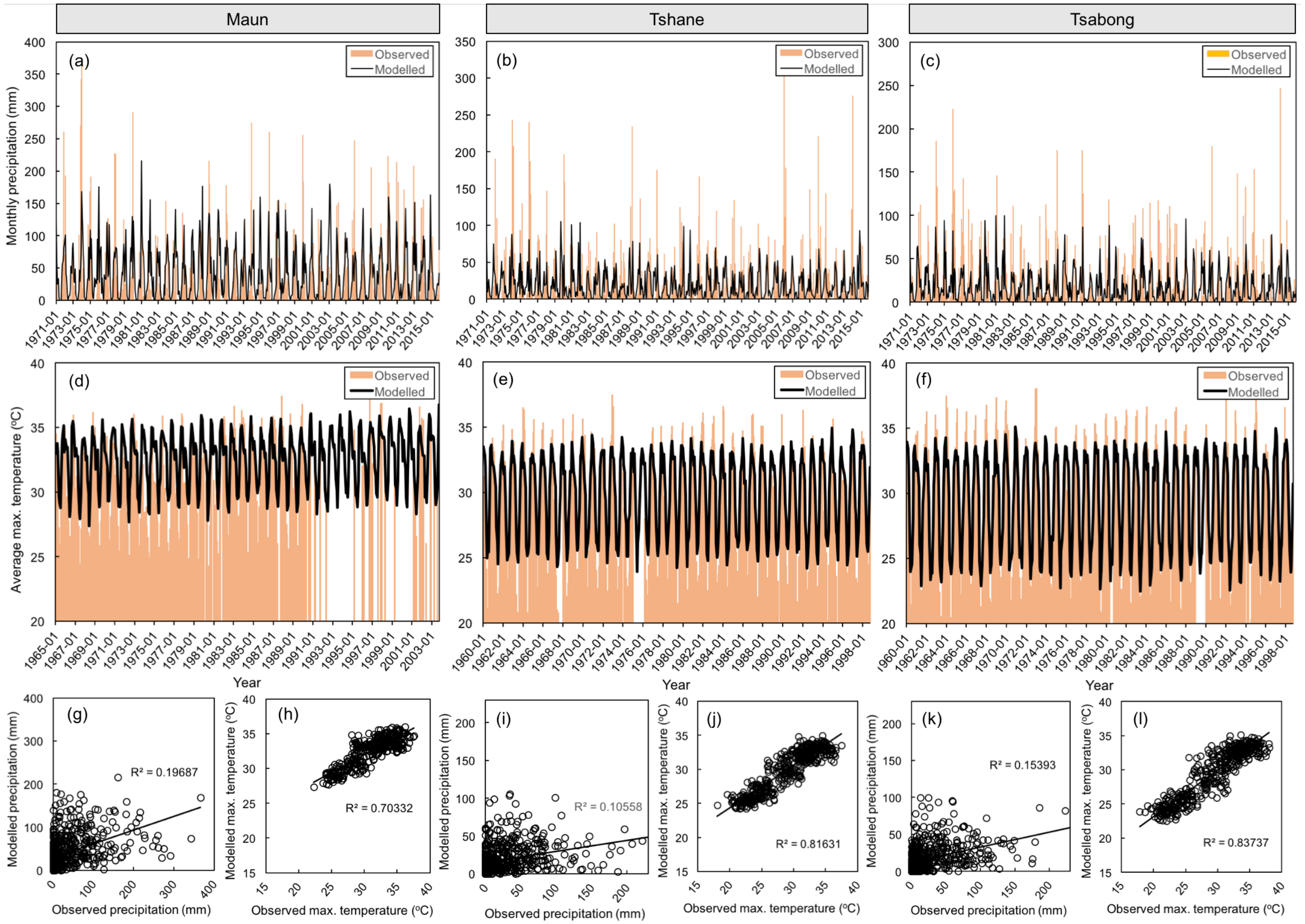

Figure 3 Modelled and observed total monthly precipitation for (a) Maun, (b) Tshane, and (c) Tsabong, over periods for which observed data were available (missing data are shown as gaps). Modelled and observed average maximum daily temperature for (d) Maun, (e) Tshane, and (f) Tsabong. Observed vs modelled temperature and precipitation for (g, h) Maun, (i,j) Tshane, (k, l) Tsabong, and (g, h) Tshane. R^2 coefficients of determination and lines of best fit are shown.

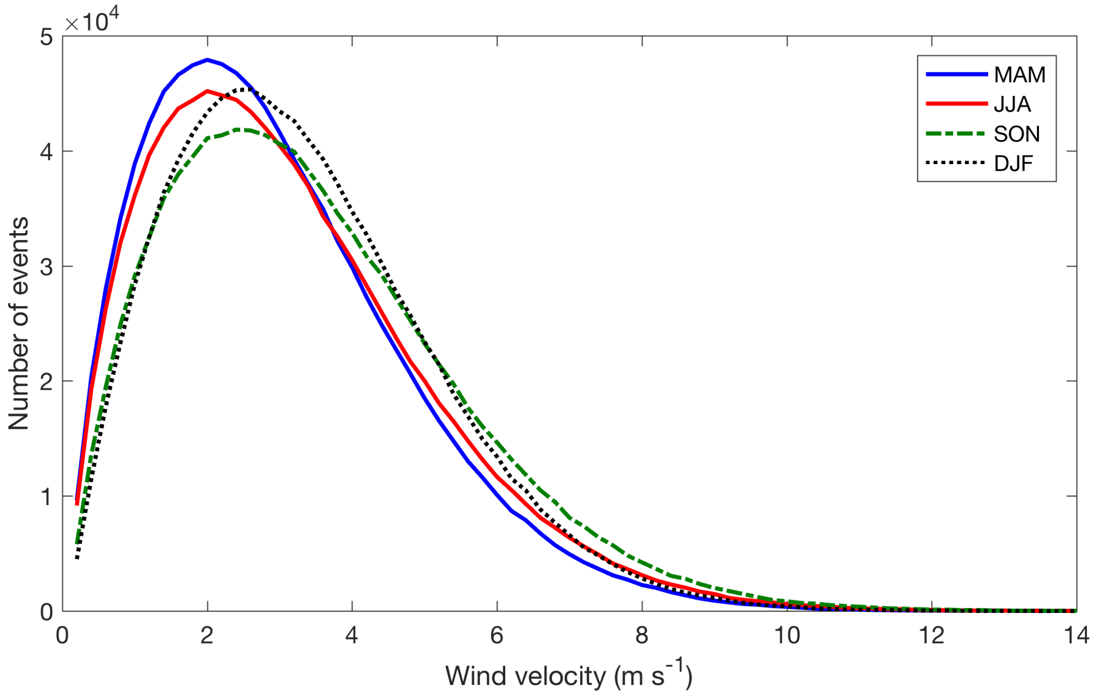

Figure 4 Weibull distributions of wind velocities recorded at Twee Rivieren at 08:00, 14:00 and 20:00 over the period 1994–2013, grouped by season.

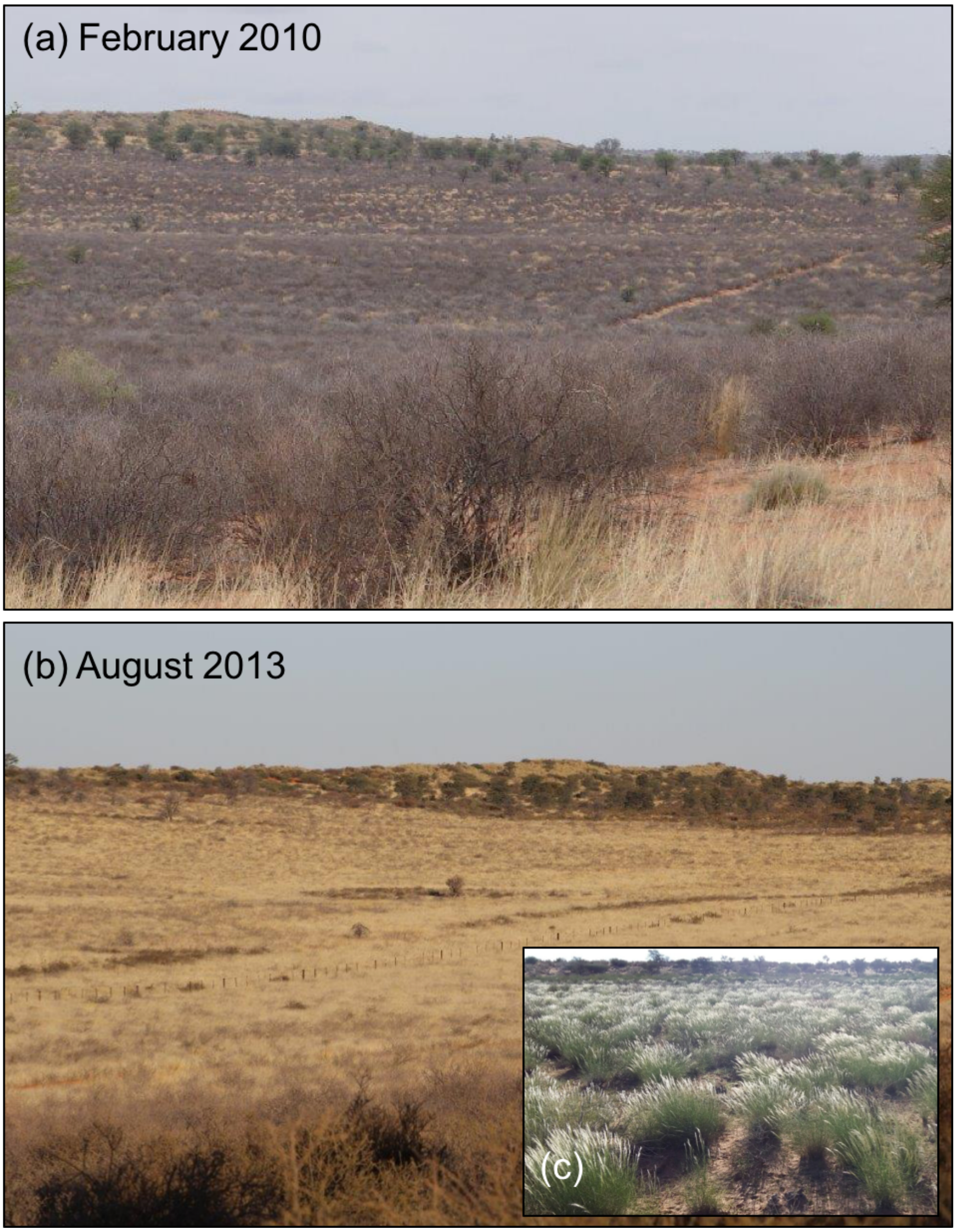

Figure 5 The replacement of woody shrubs by perennial grasses following herbicide application ('de-bushing'): (a) shrub-dominated system in early 2010, a few months after application; (b) grass-dominated system a few years later; (c) close-up of *Stipagrostis amabilis* grasses that emerged after de-bushing. Photos: P. Möller (25.50° S, 19.73° E).

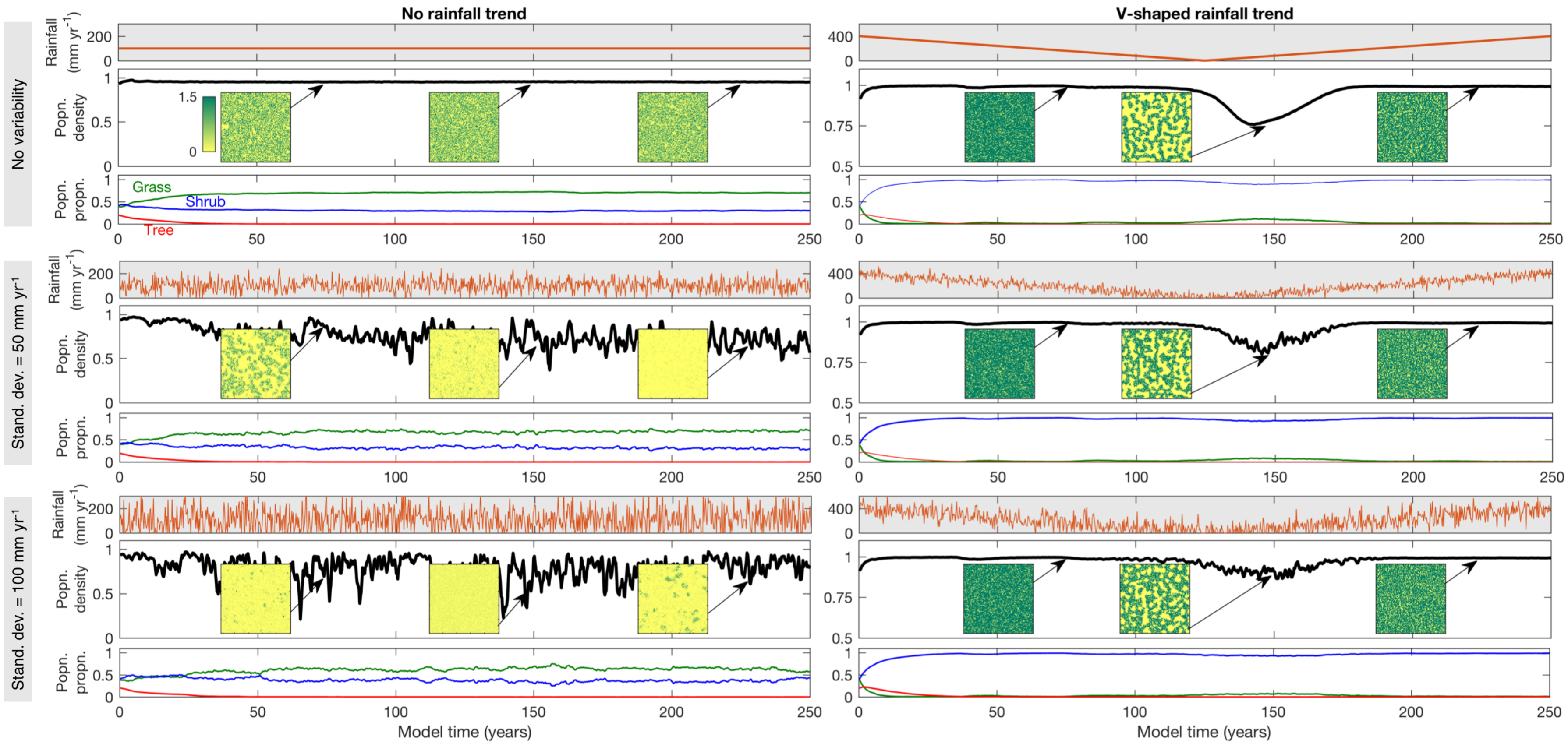

Figure 6 Effect of rainfall variability on population density and proportions of vegetation types, for two rainfall scenarios: (1) no trend in rainfall (constantly 100 mm yr^{-1}), and (2) v-shaped rainfall (decreasing from 400 mm yr^{-1} to 0 mm yr^{-1} before increasing back to 400 mm yr^{-1}). For both scenarios, three rainfall variability cases were imposed: (1) no variability, (2) normally-distributed variability with a standard deviation of 50 mm yr^{-1} (annual equivalent), and (3) normally-distributed variability with a standard deviation of 100 mm yr^{-1}. For all cases, model was run for 250 years, with vegetation updated every 3 months (the rainfall in the grey panels is given as an annual rainfall equivalent for each 3-month iteration). At the start of each run, the model was initiated with approximate equilibrium proportions of grasses, shrubs and trees (as derived by Mayaud et al. 2017). Inset panels show spatial distributions of vegetation heights across the domain at 75, 150 and 225 model years.

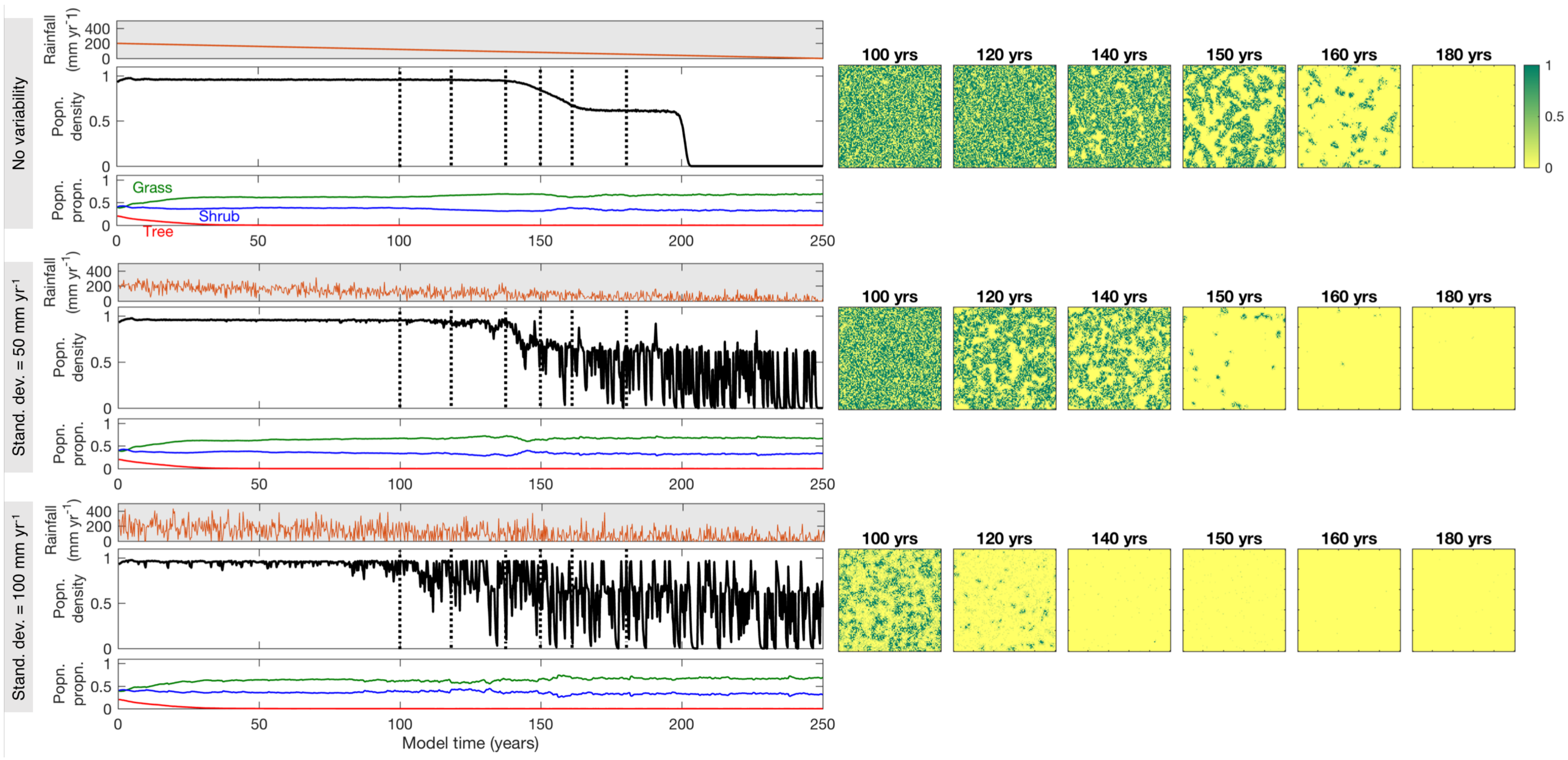

Figure 7 Effect of rainfall variability on population density and proportions of vegetation types, for a scenario of decreasing rainfall (from 200 mm yr^{-1} to 0 mm yr^{-1}. Three rainfall variability cases were imposed: (1) no variability, (2) normally-distributed variability with a standard deviation of 50 mm yr^{-1} (annual equivalent) and (3) normally-distributed variability with a standard deviation of 100 mm yr^{-1}. For all cases, model was run for 250 years, with vegetation updated every 3 months (the rainfall in the grey panels is given as an annual rainfall equivalent for each 3-month iteration). At the start of each run, the model was initiated with approximate equilibrium proportions of grasses, shrubs and trees (as derived by Mayaud et al., 2017). Panels show spatial distributions of vegetation heights at selected years spanning the observed collapse in population density (marked as dotted lines on population density graphs).

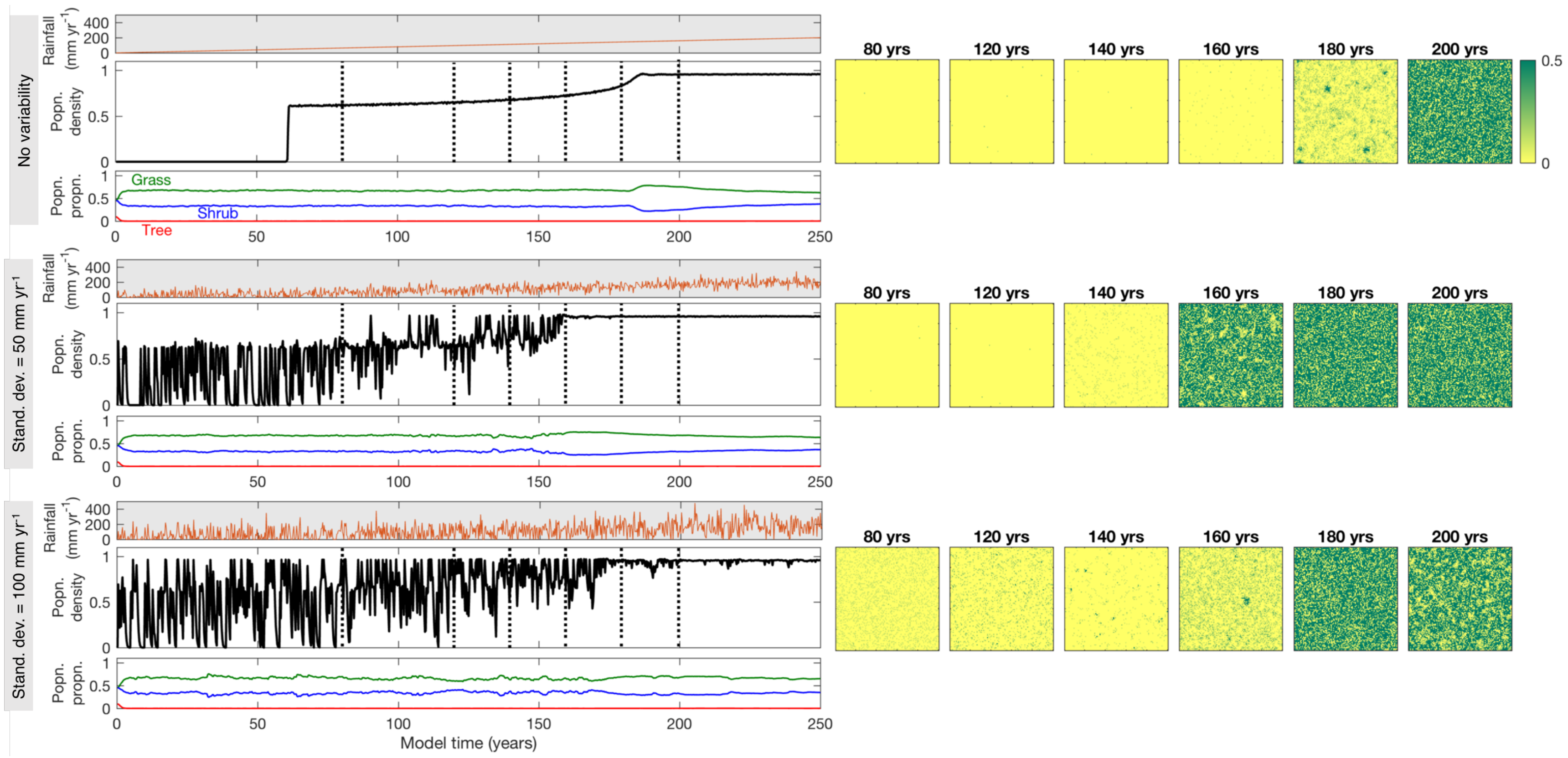

Figure 8 Effect of rainfall variability on population density and proportions of vegetation types, for a scenario of inccreasing rainfall (from 0 mm yr^{-1} to 200 mm yr^{-1}. Three rainfall variability cases were imposed: (1) no variability, (2) normally-distributed variability with a standard deviation of 50 mm yr^{-1}, and (3) normally-distributed variability with a standard deviation of 100 mm yr^{-1}. For all cases, model was run for 250 years, with vegetation updated every 3 months (the rainfall in the grey panels is given as an annual rainfall equivalent for each 3-month iteration). At the start of each run, the model was initiated with approximate equilibrium proportions of grasses, shrubs and trees (as derived by Mayaud et al., 2017). Panels show spatial distributions of vegetation heights at selected years spanning the observed collapse in population density (marked as dotted lines on population density graphs).

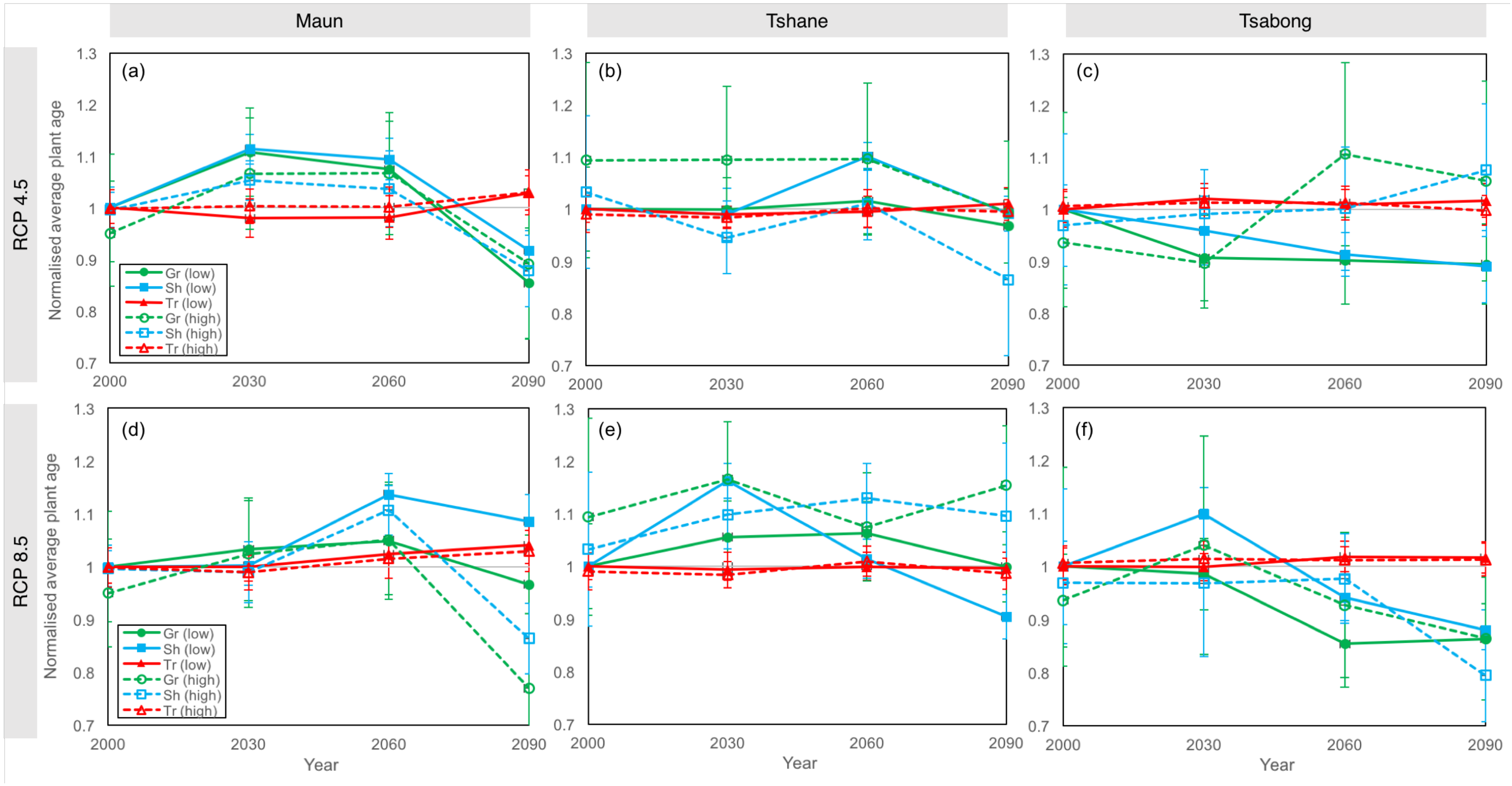

Figure 9 Equilibrium normalised annual average plant age for grasses (Gr), shrubs (Sh) and trees (Tr) at the three chosen years (2030, 2060 and 2090), normalised to the baseline year (2000, RCP 4.5), at the three study sites. Results are shown for RCP 4.5 and RCP 8.5 scenarios, in low variability (standard deviation, σ = 30 mm annual equivalent) and high variability (σ = 160 mm annual equivalent) precipitation conditions. Model was run to equilibrium for the equivalent of 200 model years, with 20 wind events every 3 months, over a grid of 150 x 150 cells. The domain was randomly initiated with 90% vegetation cover and random sediment depth. Average ages were calculated as means over the last 10 years of each run. Error bars show coefficients of variation (thus partly representing seasonal variations).

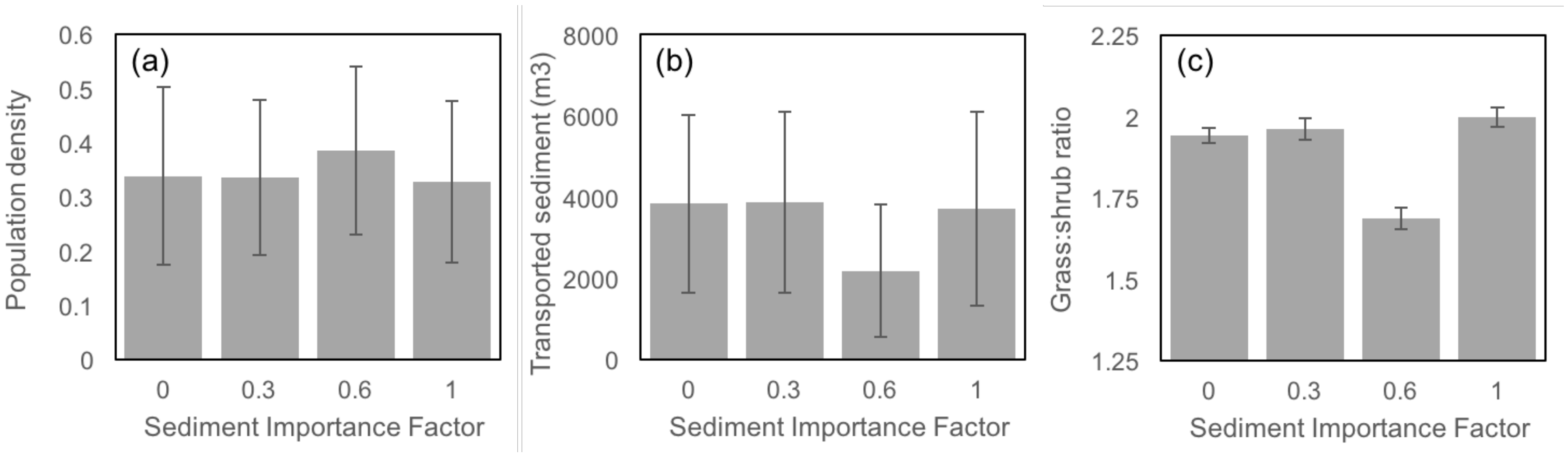

Figure 10 Effect of varying the sediment importance factor on: (a) average population density; (b) average transported sediment; (c) grass:shrub ratio, where values above unity represent grass dominance. Model was run to equilibrium for the equivalent of 100 model years, with precipitation conditions for year 2030 (RCP 4.5) at Tsabong, over a grid of 150 x 150 cells. 10 wind events of constant wind velocity (7 m s^{-1}; threshold for entrainment = 5 m s^{-1}) occurred every 3 months. The domain was randomly initiated with 90% vegetation cover and random sediment depth. Average statistics were calculated as means over the last 10 years of each run. Error bars show standard deviations (thus partly representing seasonal variations).

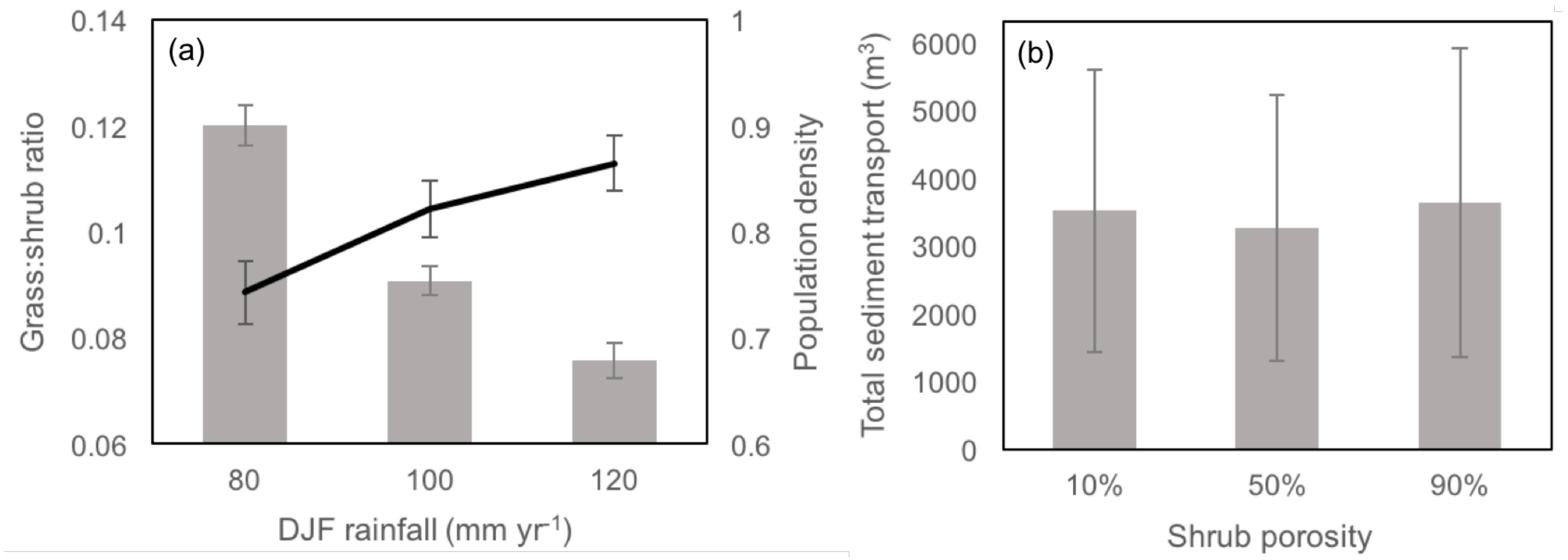

Figure 11 (a) Impact of varying summer (DJF) rainfall on grass:shrub ratios (grey bars) and population density (black line). Model was run to equilibrium for the equivalent of 100 model years, over a grid of 150 x 150 cells, with MAM, JJA and SON precipitation conditions for year 2030 (RCP 4.5) at Tsabong, and DJF rainfall was varied between 80 mm yr^{-1} and 120 mm yr^{-1}. The domain was randomly initiated with 90% vegetation cover and no sediment transport was simulated; (b) Impact of varying fixed shrub porosity on total sediment transport across the domain. Model was run to equilibrium for the equivalent of 100 model years, with precipitation conditions for year 2030 (RCP 4.5) at Tsabong, over a grid of 150 x 150 cells. 10 wind events of constant wind velocity (7 m s^{-1}; threshold for entrainment = 5 m s^{-1}) occurred every 3 months. The domain was randomly initiated with 90% vegetation cover and random sediment depth. In both figures, error bars show standard deviations (thus partly representing seasonal variations), and average statistics were calculated as means over the last 10 years of each run.

Tables

Months	Number of usable data points	Weibull shape parameter (β)	Weibull scale parameter (η)
MAM	5378	1.67	3.24
JJA	5232	1.65	3.42
SON	5183	1.75	3.78
DJF	5188	1.87	3.64

Table 2 Number of usable data points (over the period 1994–2013), and maximum likelihood estimates of the 2-parameter Weibull distribution, for each month grouping.

Experiment (related figure)	N° of repeats	RCP	Modelled years	Veg. update interval	Wind freq.	Grid length (m)	Cell size (m)
Equilibrium runs (Fig. 3, Supp. Fig. 8)	Equilibrium (200 yrs)	4.5, 8.5	2000, 2030, 2060, 2090	3 months	20 events / 3 months	150	1
Transient runs (Fig. 4)	10 per site/RCP	4.5, 8.5	1960 – 2100	3 months	20 events / 3 months	150	1
Fire/grazing runs (Fig. 5, Fig. 6)	3 per site/RCP	4.5	1960–2100	3 months	20 events / 3 months	150	1
Rainfall variability runs (Supp. Fig. 5, Supp. Fig. 6, Supp. Fig. 7)	Single (250 yrs)	-	-	3 months	-	100	1

Table 1 Summary of model state parameters for the experiments presented in this study.
